# Evaluation of phenotypic and behavioral toxicity of micro- and nano-plastic polystyrene particles in larval zebrafish (*Danio rerio*)

**DOI:** 10.1093/toxsci/kfaf015

**Published:** 2025-02-08

**Authors:** Bailey Levesque, Sabahudin Hrapovic, Fabrice Berrué, Anja Vogt, Lee D Ellis, Ludovic Hermabessiere

**Affiliations:** National Research Council Canada, Aquatic and Crop Resource Development Research Centre, Halifax, NS B3H 3Z1, Canada; National Research Council Canada, Aquatic and Crop Resource Development Research Centre, Montréal, QC H4P 2R2, Canada; National Research Council Canada, Aquatic and Crop Resource Development Research Centre, Halifax, NS B3H 3Z1, Canada; National Research Council Canada, Aquatic and Crop Resource Development Research Centre, Charlottetown, PEI C1A 4P3, Canada; National Research Council Canada, Aquatic and Crop Resource Development Research Centre, Halifax, NS B3H 3Z1, Canada; National Research Council Canada, Aquatic and Crop Resource Development Research Centre, Halifax, NS B3H 3Z1, Canada

**Keywords:** accumulation, tissue localization, toxicity, ZET, stress response

## Abstract

Plastic particles have been found in all environments and it is necessary to understand the risks these particles pose in, and to, the environment. The objectives of this work were to understand the toxic effects of varying size and concentration of polystyrene (PS) micro- and nano-plastics in zebrafish embryos and their fate within the larvae. In this work, larval zebrafish (*Danio rerio*) were exposed to six sizes (0.05, 0.25, 0.53, 2.1, 6.02, and 10.2 µm diameter) and concentrations (0.0005 to 0.2 µg/µL) of PS micro/nanoplastics particles. The zebrafish embryo toxicity (ZET) and the general and behavioral toxicity (GBT) assays were used to determine particle toxicity in embryos. Behavioral analysis was performed and micro/nanoplastics uptake and organ distribution were assessed. Phenotypic and behavioral toxicity was observed in all exposures with the exception of 0.25 µm particle-exposed larvae. Significant phenotypic toxicity was seen at the highest tested exposure concentration, with some sizes showing potential recovery as time increased in the assay. Behavioral analysis demonstrated a decrease in baseline activity across all micro- and nano-plastic sizes. Significant increases in light–dark responses were recorded in ZET assays of smaller-sized particles and no significant effects were observed at larger sizes. Significant decreases in this response were reported in the GBT assays of all tested sizes with the exception of the 0.05-µm particles. These assays demonstrate the general, developmental, and neurotoxicity of micro/nanoplastics to a model organism, which can be used to infer individual and population-level effects of exposure.

## Graphical Abstract

**Figure:**
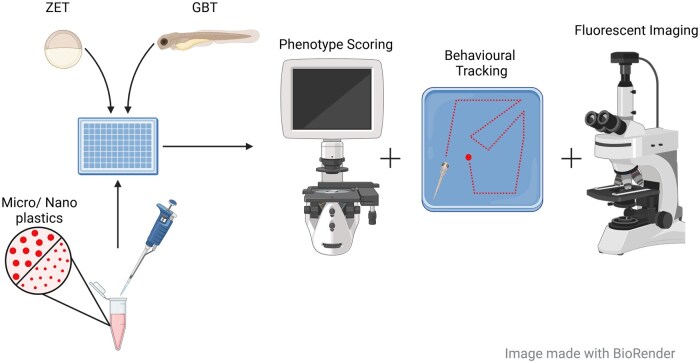

Plastic debris has been accumulating in the environment since the start of large-scale plastic production in the 1950s (GESAMP 2019), and in recent years has been detected globally in all environments (Rochman and Hoellein 2020). When identified in the environment, plastic particles are classified based on size: Macroplastic (>5 mm), microplastic (1 to 5,000 µm), and nanoplastic (<1 µm) (Gigault et al. 2018). Plastic debris enters the environment through various pathways (Li et al. 2023) and once environmentally present, microplastics and macroplastics may be mechanically (Ahrendt et al. 2020), biologically (Casillas et al. 2023), or chemically (Andrady 2011) degraded into smaller particles, i.e. nanoplastic. Such degradation from microplastic to nanoplastic has recently been demonstrated in the laboratory (Dawson et al. 2018; Khan and Ali 2023).

Due to the increased prevalence of plastic particles in the environment, published literature within this field of research has increased steadily (Casillas et al. 2023) and many studies have shown the ingestion of microplastic by organisms in the environment (Al-Sid-Cheikh et al. 2018; GESAMP 2019; Ahrendt et al. 2020; Casillas et al. 2023; Carrillo-Barragán et al. 2024), including in fish (Limonta et al. 2019; Clark et al. 2023; Zhou et al. 2023). Interaction of micro- and nano-plastics with organisms can result in deleterious effects. For example, polystyrene (PS) particles have been shown to induce vascular malformation (Dai et al. 2023), morphological abnormalities (De Marco et al. 2022), oxidative stress (De Marco et al. 2022), and effects on locomotor activity (Chen et al. 2017, 2020) in zebrafish (*Danio rerio*). It has been suggested that particle transfer between organs and food dilution are the main drivers of toxicity to organisms (de Ruijter et al. 2020; Mehinto et al. 2022). Risk assessments for microplastics in the marine environment and in humans have recently been published highlighting that more data are required to improve estimates of the risk posed by micro- and nano-plastics (Coffin et al. 2022; Mehinto et al. 2022).

Micro- and nano-plastics have previously been shown to negatively affect both larval and adult zebrafish including effects on development (De Marco et al. 2022), gene transcription (Limonta et al. 2019), and behavior (Chen et al. 2020). PS 10 µm particles were shown to induce developmental abnormalities in larvae including spinal curvature, blood pooling, column deformation, and edema (De Marco et al. 2022). In addition, the immune response of adult zebrafish can be affected by exposure to PS particles through the upregulation of genes associated with the activation of an immune response, antiviral defense, and antimicrobial response following a 20-day exposure (Limonta et al. 2019). PS microparticles have also been shown to influence zebrafish behavior, specifically increasing the activity level in adult zebrafish after exposure to 5 µm particles (Chen et al. 2020).

In the present study, zebrafish larvae were used to determine developmental, general, and neuro-toxicity of various sizes of PS micro- and nano-plastic particles through waterborne exposure. The objectives of the study were to (i) understand the effects of particle size of micro- and nano-plastics on zebrafish development and behavior, (ii) understand how size influences particle distribution within the larvae, and (iii) determine the toxicity of these particles. Six particle sizes were tested as it has been observed that size can affect particle toxicity (Zhou et al. 2023). It was hypothesized that smaller-sized particles would be more toxic than larger particles as seen through increased incidence of recorded toxic phenotypes and alterations of larval behavior. In addition, it was predicted that smaller particles (nanoplastics) would cross biological barriers and would be distributed within organs of the zebrafish larvae, whereas larger particles (microplastics) would only be found in the gastrointestinal tract of the larvae.

## Materials and methods

### Materials

PS particles were purchased from Spherotech (Lake Forest, IL, United States) at a concentration of 1% w/v and a density of 1.05 g/mL. Particles were composed of Nile Red dye polymerized with styrene to ensure no fluorophore leaching. Stock solutions were kept at room temperature in the dark. Working dilutions were prepared immediately prior to the commencement of both zebrafish embryo toxicity (ZET) and general and behavioral toxicity (GBT) assays described below (Achenbach et al. 2020). Working solutions were filtered and fluorescence intensity was determined to confirm the absence of leaching throughout the experiment. Exposure concentrations included 0.2, 0.1, 0.07, 0.05, 0.02, 0.001, and 0.0005 µg/µL (200, 100, 70, 50, 20, 1, and 0.5 ppm). Product numbers, size range, and mean particle diameter are included in Table S1.

### Particle characterization

Transmission electron microscopy (TEM, HITACHI H-7500, Japan) equipped with bottom-mounted AMT NanoSprint 12MP camera and operating at 80 kV in high-contrast mode, was performed on samples 0× to 50× diluted in Millipore water (Fig. S1). TEM grids (copper 200 mesh, 12-25 nm carbon supported, Ted Pella Inc.) were freshly glow-discharged using EMS GloQube-D, Dual chamber glow discharge system (Electron Microscopy Sciences, PA) in negative mode with plasma current of 25 mA for 45 s. Grids were floated on 20 μL sample aliquots on Parafilm for 3 min. Excess droplets were subsequently wicked away from the edge of the grid with the filter paper strips (Whatman 541). The grid was then rinsed with droplets of double distilled water. Finally, the grid was dried at ambient conditions for 2 h and used for TEM analysis.

Dynamic light scattering (DLS) was used to determine the average particle size and the zeta potential of samples, typically 100× diluted in Millipore water using a Zetasizer Nano-ZS (Malvern Instruments, Malvern, United Kingdom). The DLS analyses were done in triplicate and the average was reported (Table S2). DLS data were used to estimate individual particle mass and the number of particles per unit volume equivalent to each exposure concentration (Table S3).

### Animal husbandry

Zebrafish (*Danio rerio*) were housed in accordance with the Canadian Council on Animal Care guidelines (CCAC 2020) and standard animal care protocols and bred onsite. The AB/Tubingen zebrafish adults, embryos, and larvae were housed in a recirculating Tecniplast aquatic system (West Chester, PA, United States) with a 14:10 h light:dark cycle. The system maintained a constant temperature of 28.5 ± 1 °C and pH of 7.0 to 7.5. Following breeding of adult zebrafish, embryos were collected and incubated (Fisherbrand Isotemp Model 146E, Fisher Scientific, NH, United States) in E3 (5 mM NaCl, 0.17 mM KCl, 0.33 mM CaCl_2_-2H_2_O, 0.33 mM MgSO_4_-7H_2_O) media until experimentation. At 6 hpf (hours postfertilization), embryos were used for experimentation in ZET assays or placed in Aquatic Habitats (Pentair Aquatic Eco-Systems, Apopka, FL, United States) mesh-bottom nursery baskets on the recirculation system until 72 hpf for later use in GBT assays. After the completion of experimental protocols larvae were euthanized with a lethal dose of tricaine methanesulfonate (3-amino benzoic acid ethyl ester, stock solution = 4 mg/mL in water; Sigma-Aldrich, MO, United States) buffered with sodium bicarbonate (stock solution = 8 mg/mL).

### Confirmation of uptake

Larvae were prepared according to the ZET and GBT protocols below and imaged at time points of 72, 96, 120 and 96, 120 hpf, respectively, to confirm particle uptake. At each time point, larvae were removed from the well plate, using a transfer pipette, and placed into a 74-mm Netwell (Corning, United States). The exposure media was filtered through the Netwell and larvae were then washed twice in E3 (5 mM NaCl, 0.17 mM KCl, 0.33 mM CaCl_2_-2H_2_O, 0.33 mM MgSO_4_-7H_2_O) to remove any plastic particles that may have adhered to the outside of the body. Following washing, larvae were placed into a 6-well PS plate containing 2.5 mL of a Tricaine methanesulfonate anesthetic and E3 solution at 168 µg/mL.

Larvae were left in the anesthetic solution for 10 min prior to being individually loaded into a Vertebrate Automated Screening Technology (VAST) BioImager (Union Biometrica, Holliston, MA, United States). Using the VAST, in combination with a Nikon AZ100 microscope (Nikon Instruments Inc., NY, United States), dorsal, ventral, and lateral views of larvae were recorded using both brightfield and fluorescent (TRIT-C filter) views.

### Phenotypic toxicity: Zebrafish Embryo Toxicity (ZET) assay

To determine developmental toxicity using the ZET assay and subsequent effects on behavior, fertilized zebrafish embryos were collected at 6 ± 0.5 hpf and transferred individually into wells of a square well flat-bottomed 96-well PS plate in a total volume of 270 µL of HEPES-buffered E3 (HE3) media (5 mM NaCl, 0.17 mM KCl, 0.33 mM CaCl_2_-2H_2_O, 0.33 mM MgSO_4_-7H_2_O, 10 mM HEPES, pH 7.2). This assay was based on the OECD protocol 236 (OECD 2013). Larvae were then inoculated to a final volume of 300 µL with concentrated microplastic solutions reaching desired nominal concentrations (0.0005, 0.001, 0.02, 0.05, 0.07, 0.1, and 0.2 µg/µL). Concentrations were calculated based on mass. Following inoculation, plates were sealed with ThermoSeal RTS clear-transparent film (Excel Scientific, Victorville, CA, United States) to prevent evaporation and incubated at 28.5 °C in a 14:10 h light:dark cycle (light intensity 3 to 5 µmol/m^2^/s). Twelve larvae were exposed per concentration of microplastic along with 12 negative control larvae (HE3 only). Exposures were done in triplicate. Larvae were scored visually using an inverted microscope (Invitrogen EVOS XL Core Imaging System, Thermo Fisher Scientific, MA, United States) at 72, 96, and 120 ± 0.5 hpf for mortality and phenotypic malformations. Larvae were classified as dead, if there was no observable heartbeat. At 120 hpf, plates were immediately used for behavioral analysis.

### Phenotypic toxicity: General and Behavioral Toxicity (GBT) assay

The GBT analysis is an expansion on the OECD 236 model (OECD 2013), where larvae are exposed to a test compound after body patterning is largely complete. This is an established juvenile model (Achenbach et al. 2020) and has been used here to determine general toxicity. Fertilized embryos were kept on the Tecniplast system until 72 hpf at which point they were individually transferred to 96-well flat-bottomed well plates with 270 µL of HE3. Immediately following transfer, larvae were inoculated with 30 µL of a concentrated microplastic solution to reach experimental concentrations (0.0005, 0.001, 0.02, 0.05, 0.07, 0.1, and 0.2 µg/µL). The plates of larvae were incubated under the same conditions as described in the ZET assay above. Mortality and phenotypic malformations were recorded at 96 and 120±0.5 hpf using an inverted microscope (Invitrogen EVOS XL Core Imaging System, Thermo Fisher Scientific, MA, United States). At 120 hpf, larvae were used for behavioral analysis.

### Behavioral analysis

For both ZET and GBT assays, plates at 120 hpf were placed into a Noldus Daniovision Behavioural Tracking instrument (Noldus, Leesburg, VA, United States) without the sealing film. Movement of larvae was recorded for a 1 h period, during which larvae were exposed to 30 min of light (15 µmol/m^2^/s) followed by three 5-min alternating dark and light periods. Larvae were kept at a constant temperature of 28.5 °C throughout the assay. After behavioral tracking, larvae were scored for mortality and phenotypic malformations.

Larvae scored as affected (dead or phenotypically malformed) were removed from downstream behavioral analysis. For any exposure concentration to be included in the behavioral analysis, there must have been at least 12 phenotypically normal larvae across all plate replicates.

### Dose curve fitting

Exposures for each tested plastic size were performed using a minimum of three replicate plates. Values of affected larvae (dead+phenotypically malformed) at 120 hpf were plotted against the log of the exposure concentration to determine LC_50_ and EC_50_, respectively, using a line of best fit with variable slope (four parameters). All curves were constrained to fit to 0. If 100% lethality or 100% affected was observed at the highest tested exposure concentrations, the curves were also constrained to fit to 100. If 100% lethality or affected were not seen, no observable effect concentration (NOEC) and lowest observable effect concentration (LOEC) values were determined in the place of EC_50_ and LC_50_ values. In this work, the definition of the LOEC and NOEC given by the United States Environmental Protection Agency (US EPA) was used. The LOEC is described as follows “The lowest concentration of a material used in a toxicity test that has a statistically significant adverse effect on the exposed population of test organisms compared with the controls.,” whereas the NOEC is defined as “the highest concentration of a material in a toxicity test that has no statistically significant effect on the exposed population of test organisms compared with the controls.” (United States Environmental Protection Agency 2024).

### Statistical analysis

To study the toxic effects of micro- and nano-plastic concentration on zebrafish larvae, the occurrence of affected larvae (dead and phenotypically malformed) as well as behavioral responses were analyzed using one-way ANOVAs. Normality was assessed using the Shapiro–Wilks test and visually confirmed with a QQplot. Phenotypic and behavioral data did not meet the conditions of normality and analyses were performed using the nonparametric Kruskal–Wallis test and were followed by a Dunn’s multiple comparisons test if significant effects were found. Dunn’s multiple comparison tests were used to explore statistical differences between each nominal concentration and the controls. Significance was determined at *P* < 0.05. Phenotypic toxicity data were analyzed using R (Version 4.3.1) (RC Team 2013) and figures were created using the ggplot2 package (Version 3.4.4) (Wickham et al. 2016). Asterisks denoting significance were added using Gimp 2 software (Version 2.10.38). Behavioral toxicity data analysis and figure creation were completed using both R (using ggplot2) and Graphpad Prism (version 10.1.0 [316]). Response to light–dark transitions were calculated by subtracting the distance traveled during the preceding 5 min light period prior to the dark period from the distance traveled in the dark period.

## Results

Control media and filtered microplastic samples were compared and no statistically significant differences were found, thus confirming Nile Red leaching did not occur throughout the assays (Fig. S2).

### Confirmation of uptake

**Fig. 1.**
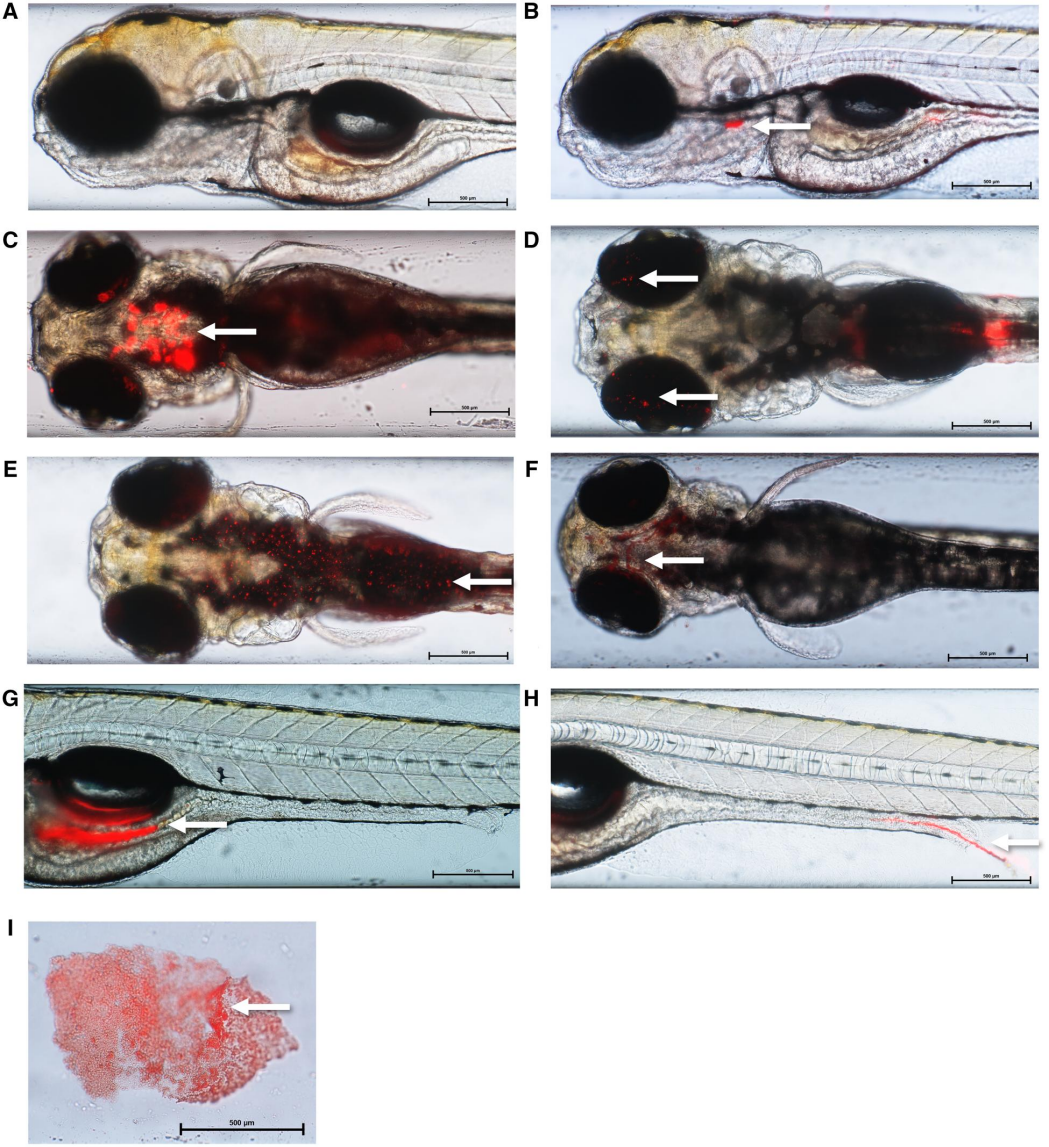
Representative images of Nile Red labeled PS micro- and nano-plastic particles in larval zebrafish demonstrating accumulation in the esophagus (B), mouth (C), eyes (D), dermal tissue (E), gills (F), gastrointestinal tract (G), hatched chorion (I), and excretion (H) as well as a control (A). White arrows point to the areas of plastic accumulation. Larvae were imaged at time points of 72 hpf (F, I), 96 hpf (A, B, C, E), and 120 (D, G, H) following ZET (B, C, F, G, I) or GBT (A, D, E, H) assays. Exposure concentrations were 0.02 (E), 0.05 (D), 0.1 (H), and 0.2 µg/µL (B, C, F, G, I). The above larvae were exposed to microplastic sizes of 0.25 µm (B, E, F, G, I), 0.53 µm (D, H), and 2.1 µm (C). Images are composed of a TRIT-C filter overlay on a brightfield image taken with a 4× objective and 2× magnification using a Nikon AZ100.

Microplastic uptake was visually confirmed for all sizes of micro- and nano-plastic tested. Plastic particles accumulated in the gastrointestinal tract for all sizes (Fig. 1) and accumulation patterns for each size are summarized in Table S4.

Imaging of larvae exposed to 0.05 µm particles confirmed particle uptake at all time points during both ZET and GBT assays at concentrations of 0.02, 0.05, 0.07, 0.1, and 0.2 µg/µL. Particles were identified in the gastrointestinal tract, mouth, esophagus, gills, dermal tissue, and on the eyes.

Areas of accumulation of 0.25 µm particles included the exterior of the chorion, mouth, gills, esophagus, gastrointestinal tract, dermal tissue, and eyes. Presence of particles was recorded during the ZET assay at 72 hpf (0.05, 0.07, 0.1, and 0.2 µg/µL), 96 hpf (0.2 µg/µL), and 120 hpf (0.05, 0.07, 0.1, and 0.2 µg/µL). During the GBT assay, particle uptake was confirmed at 96 hpf at concentrations of 0.02, 0.05, 0.07, 0.1, and 0.2 µg/µL and at 120 hpf at concentrations of 0.05, 0.07, 0.1, and 0.2 µg/µL.

The 0.53-µm particles were observed at all ZET assay time points at concentrations of 0.02, 0.05, 0.07, 0.1, and 0.2 µg/µL. The GBT assay confirmed uptake at 96 hpf and at 120 hpf at concentrations of 0.02, 0.05, 0.07, 0.1, and 0.2 µg/µL. These particles were seen in the gills, gastrointestinal tract, esophagus, and on the eyes.

Uptake of 2.1 µm particles was seen in the mouth, esophagus, and gastrointestinal tract. This was confirmed during the ZET assay at concentrations of 0.05, 0.07, 0.1, and 0.2 µg/µL at 72 hpf and at concentrations of 0.02, 0.05, 0.07, 0.1, and 0.2 µg/µL at 96 hpf. No particles were visualized in the larvae at 120 hpf at all concentrations during this assay. During the GBT assay, 2.1 µm particles were observed in the gastrointestinal tract, esophagus, and mouth at 96 hpf at exposure concentrations of 0.02, 0.07, 0.1, and 0.2 µg/µL. Particles were also present in the same organs at 120 hpf at concentrations of 0.0005, 0.02, 0.05, 0.07, 0.1, and 0.2 µg/µL.

Larvae exposed to 6.02 µm microplastics saw particle accumulation in the esophagus, gastrointestinal tract, and mouth. Uptake was confirmed during the ZET and GBT assays at time points of 72 hpf (0.1 and 0.2 µg/µL), 96 hpf (0.02, 0.05, 0.07, 0.1, and 0.2 µg/µL), 120 hpf (0.02, 0.07, 0.1, and 0.2 µg/µL), and 96 hpf (0.02 and 0.2 µg/µL) and 120 hpf (0.07, 0.1, and 0.2 µg/µL), respectively.

When exposed to 10.2 µm microplastics, particles were taken up during the ZET assay at all three time points. At 72 hpf uptake was only seen at 0.2 µg/µL in the esophagus. At 96 hpf particles were seen in the esophagus and gastrointestinal tract at 0.07, 0.1, and 0.2 µg/µL. At the last time point of the assay, 120 hpf, particles were observed in the esophagus and mouth when exposed to concentrations of 0.05, 0.1, and 0.2 µg/µL. During the GBT assay particles were also recorded in larval tissue at 96 hpf at 0.1 and 0.2 µg/µL and at 120 hpf at exposure concentrations of 0.02, 0.05, 0.07, 0.1, and 0.2 µg/µL. During the GBT, particles were seen to accumulate in the esophagus, mouth, and gastrointestinal tract.

### Phenotypic toxicity: ZET and GBT assays

**Fig. 2.**
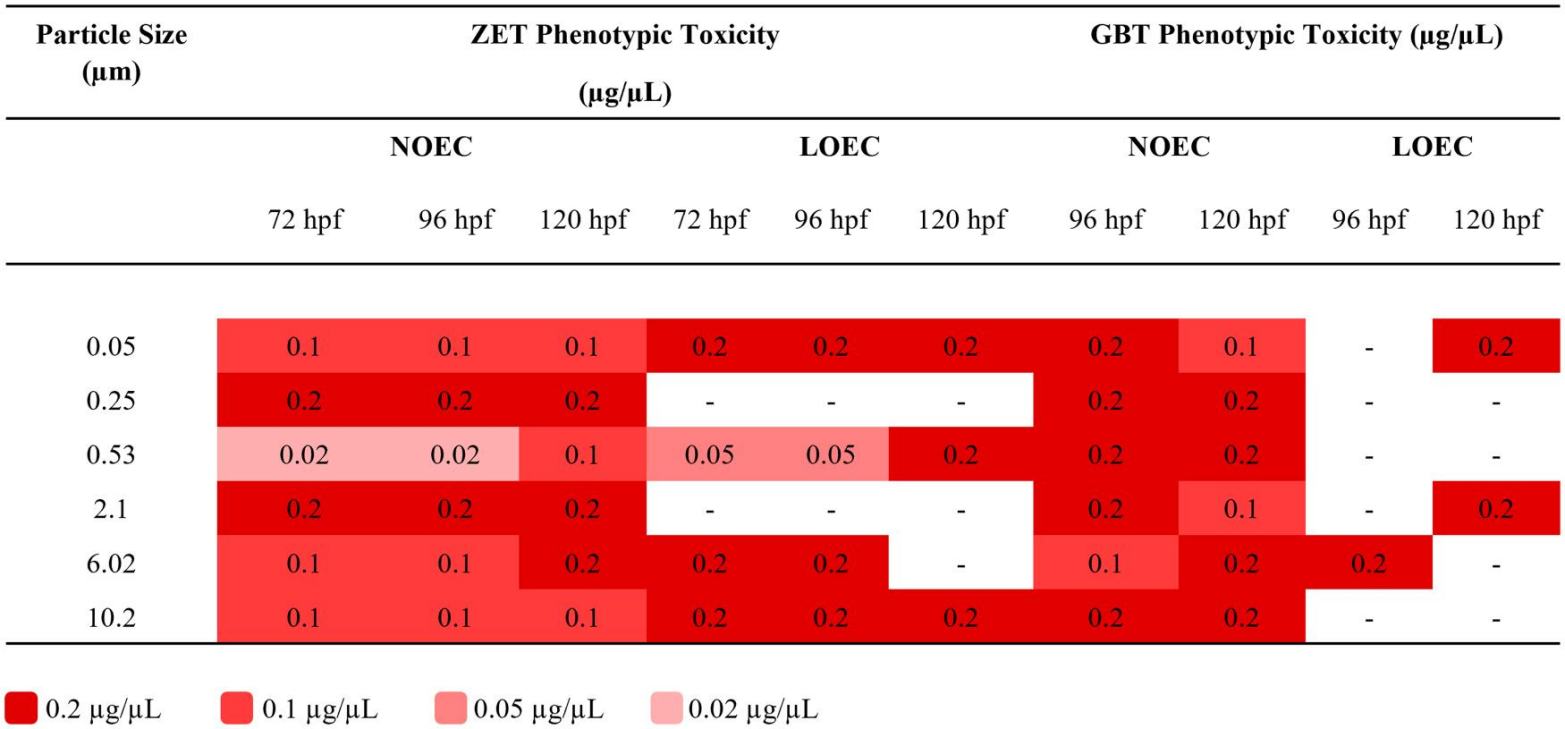
Summary of the NOEC and LOEC values at 120 hpf for phenotypic toxicity following ZET and GBT exposures of larvae to six different sizes of microplastic.

Significant toxic effects were seen in both the phenotypic and behavioral portions of the ZET and GBT assays for all tested microplastic sizes, with the exception of 0.25 µm, where no significant effect was seen, and 0.53 µm where no significant effect was seen during the GBT phenotypic assay. As exposure to plastic particles did not result in 100% mortality, for any size, NOEC and LOEC values were identified in the place of EC_50_ and LC_50_ values (Fig. 2).

Larvae exposed to 0.05 µm nanoplastics were significantly affected when exposed to 0.2 µg/µL in comparison with control larvae at all time points during the ZET assays (Fig. S3). At 120 hpf during the ZET assay, the NOEC was 0.01 µg/µL and the LOEC was 0.2 µg/µL (Fig. 2). Unhatched larvae were the most common phenotype recorded at 72 hpf during the ZET assay. This changed to light color at 96 hpf and scoliosis and no swim bladder at 120 hpf (Table S5).

**Fig. 3.**
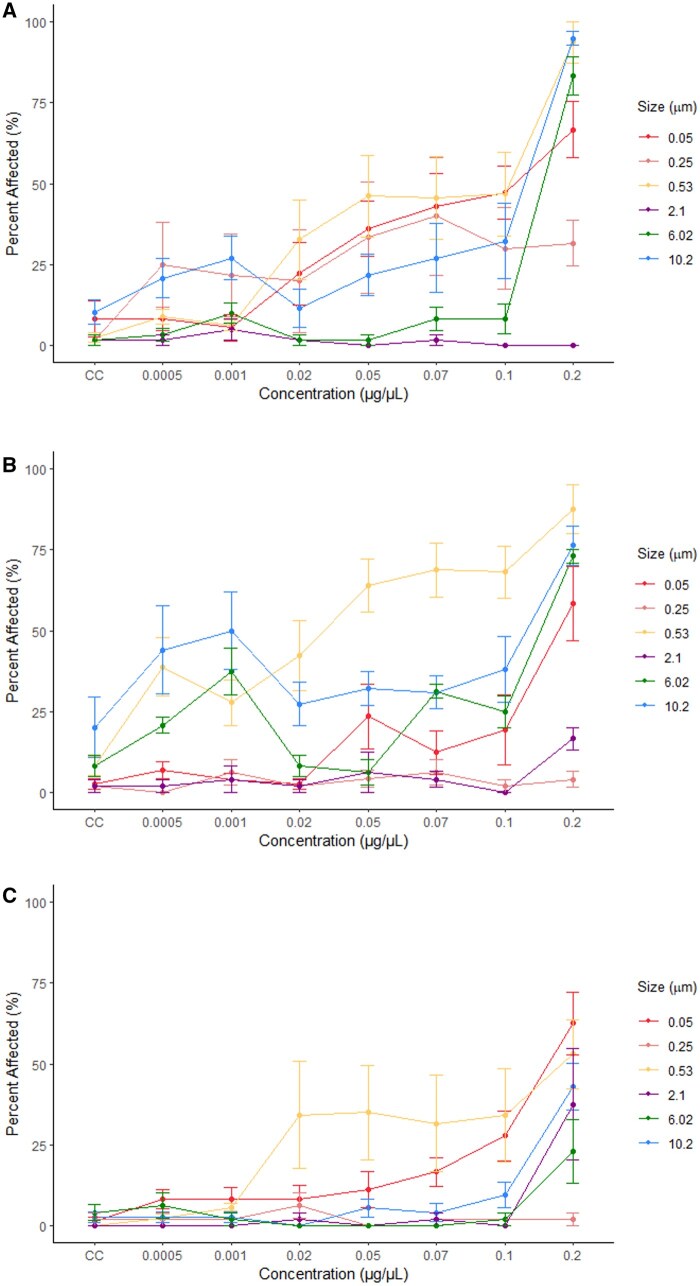
Mean percent and standard error of the mean of zebrafish larvae classified as affected following exposure to microplastics at seven exposure concentrations and a negative control (CC). Data are shown for larvae at 72 (A) (*n* = 4,031), 96 (B) (*n* = 3,551), and 120 (C) (*n* = 3,264) hpf during a ZET assay.

Exposure to 0.25 µm microplastic particles did not result in any significant differences in the percent of larvae classified as affected compared to the control in the ZET assay (Fig. S4) resulting in a NOEC value of 0.2 µg/µL and no LOEC value (Fig. 2). It is interesting to note that zebrafish larvae appeared more affected at earlier timepoints (72 hpf) compared to later timepoints (120 hpf) (Fig. 3). The most commonly recorded phenotypes were small head at 72 hpf, light color at 96 hpf, and no swim bladder at 120 hpf (Table S5).

Following ZET exposure to 0.53 µm particles, multiple concentrations showed a significantly higher percentage of affected larvae compared to the control (Fig. 3). This was seen at concentrations of 0.05, 0.07, and 0.2 µg/µL at 72 hpf and 0.05, 0.07, 0.1, and 0.2 µg/µL at 96 hpf. Statistical significance was also seen at a concentration of 0.2 µg/µL at 120 hpf (Fig. S5). The ZET NOEC value at 120 hpf was 0.1 µg/µL and the LOEC was 0.2 µg/µL (Fig. 2). The most commonly observed phenotypes during the ZET assay were small head, large yolk, and no swim bladder at 72, 96, and 120 hpf, respectively (Table S5).

There was no statistical significance during the 2.1 µm ZET assay at any time point or concentration (Fig. 3 and Fig. S6) between plastic-exposed larvae and the control. This resulted in a NOEC of 0.2 µg/µL and no LOEC being determined (Fig. 2). At time points of 72, 96, and 120 hpf in the ZET assay, the most commonly recorded phenotypes were small head, scoliosis, and no swim bladder, respectively (Table S5).

When exposed to 6.02 µm microplastic particles, significant percentages of larvae were considered affected in comparison with the control at the highest concentration (0.2 µg/µL) at 72 and 96 hpf but not at 120 hpf for the ZET assay (Fig. 3 and Fig. S7). This resulted in a NOEC value of 0.1 µg/µL and a LOEC value of 0.2 µg/µL for those time points (Fig. 2). At 120 hpf, no significant differences were found between the control and the plastic-exposed larvae, therefore, no LOEC was identified and a NOEC of 0.2 µg/µL was determined (Fig. 2). The most common phenotype seen in the ZET assay at 72, 96, and 120 hpf were small head, large yolk, and no swim bladder, respectively (Table S5).

When larvae were exposed to 10.2 µm particles, a larger percentage of affected larvae were seen at 72 hpf in the ZET assay and this percentage decreased after 96 hpf (Fig. 3 and Fig. S8). However, this percentage remains significantly higher than that seen in the control throughout all time points of the ZET assay for the highest concentration of 0.2 µg/µL. From this, a NOEC value of 0.1 µg/µL and a LOEC value of 0.2 µg/µL were determined for all time points (Fig. 2). The phenotype most recorded at 72 hpf during the ZET assay was small head compared to large yolk at 96 and 120 hpf (Table S5).

**Fig. 4.**
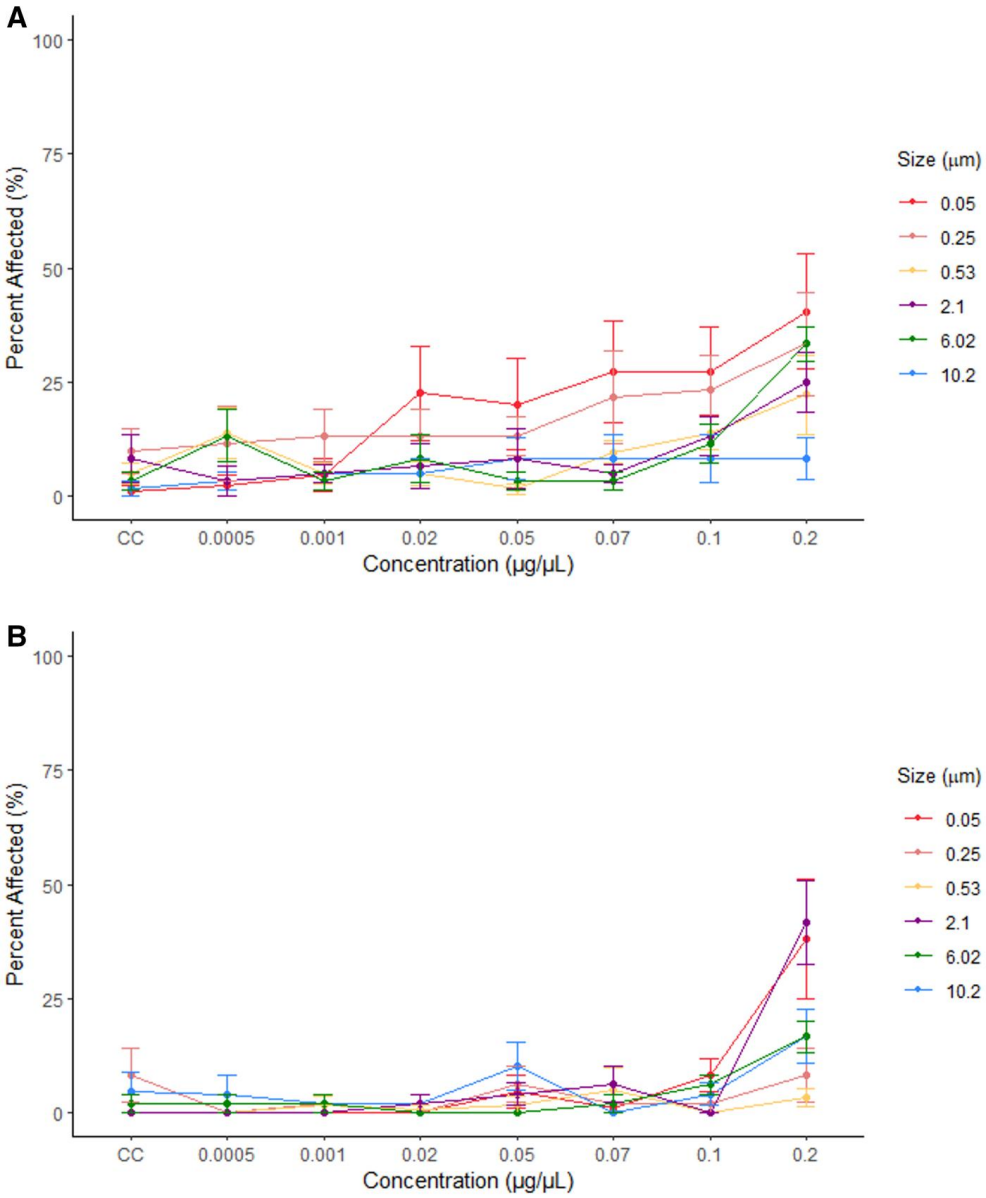
Mean percent and standard error of the mean of zebrafish larvae classified as affected following exposure to microplastics at seven exposure concentrations and a negative control (CC). Data are shown for larvae at 96 (A) (*n* = 3,358) and 120 (B) (*n* = 2,871) hpf during the GBT assay.

The GBT NOEC and LOEC values for larvae exposed to 0.05 µm plastic particles at 120 hpf were the same as those from the ZET assay, giving values of 0.1 and 0.2 µg/µL, respectively (Fig. 2). More larvae appeared to be affected at earlier timepoints (96 hpf) compared to later timepoints (120 hpf) (Fig. 4). The most recorded phenotypes in the GBT assay were light color and no swim bladder at 96 and 120 hpf, respectively (Table S5).

No significant differences were found in percent of affected larvae compared to the control following GBT exposure to 0.25 µm microplastic particles (Fig. S4). No LOEC value could be determined for this size as the NOEC was the highest tested concentration of 0.2 µg/µL. However, the percent of affected larvae appears larger earlier in the assay (96 hpf) compared to the final timepoint (120 hpf) (Fig. 4). During the GBT assay, scoliosis was the most common phenotype at both time points (Table S5).

No significant differences were found between the control and 0.53 µm microplastic exposed larvae at all time points during the GBT assay (Fig. 4). Therefore, the GBT assay NOEC value was 0.2 µg/µL and no LOEC was identified (Fig. 2). Scoliosis was the most recorded phenotype at both 96 and 120 hpf (Table S5).

Following the 2.1 µm GBT assay, there were significantly more larvae classified as affected at 0.2 µg/µL compared to the control at 120 hpf (Fig. 4 and Fig. S6). The NOEC and LOEC values for this exposure at 120 hpf were 0.1 and 0.2 µg/µL (Fig. 2). Large yolk and no swim bladder were the most observed phenotypes in the GBT assay at 96 and 120 hpf, respectively (Table S5).

During the GBT assay of larvae exposed to 6.02 µm microplastic larvae, significant differences in the percentage of affected larvae were seen between the larvae exposed to 0.2 µg/µL in comparison with the control at 96 hpf (Fig. 4 and Fig. S7). This resulted in the same NOEC and LOEC values as the ZET, 0.1 and 0.2 µg/µL, respectively (Fig. 2). As no significant differences between the control larvae and those exposed to plastic were seen at 120 hpf, the NOEC was 0.2 µg/µL and no LOEC could be determined (Fig. 2). In the GBT assay, time points of 96 and 120 hpf saw large yolk and no swim bladder as the most recorded phenotype, respectively (Table S5).

Following the GBT assay, no significant differences were found between larvae exposed to 10.2 µm particles and control larvae (Fig. 4 and Fig. S8). A NOEC of 0.2 µg/µL (Fig. 2) was identified for both time points. The GBT assay observed large yolk and scoliosis as the most common phenotypes at 96 hpf and no swim bladder as the most common at 120 hpf (Table S5).

### Behavioral analysis: ZET and GBT assays

**Fig. 5.**
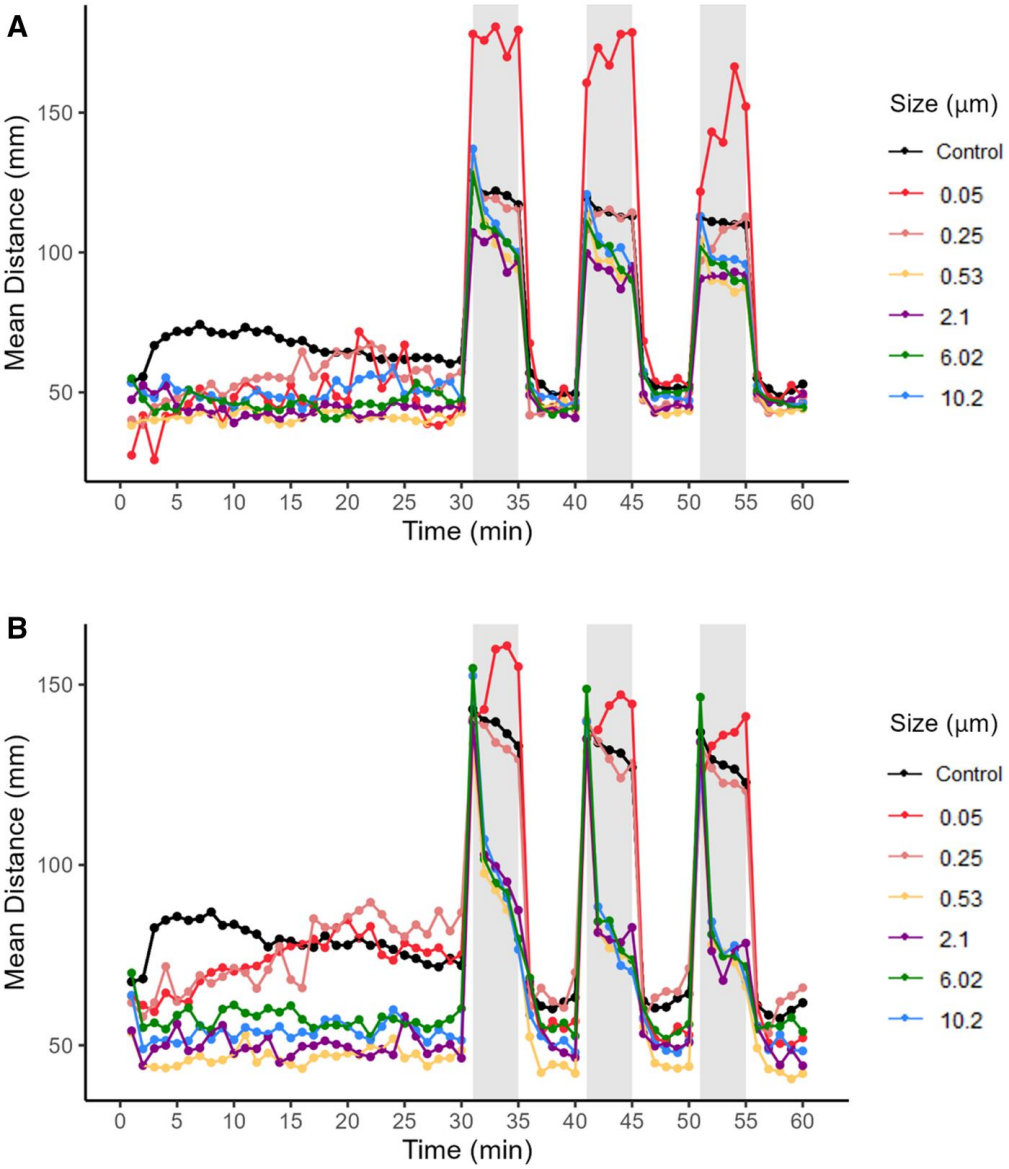
Mean distance traveled by larvae exposed to six different sizes of microplastic particles at a concentration of 0.2 µg/µL during a 60-min period following ZET (A) (Control *n* = 246 and microplastics *n* = 6 to 44) and GBT (B) (control *n* = 246 and microplastics *n* = 15 to 51) assays. Gray bars represent period, where larvae were in the dark.

This work uses assays that expand upon the OECD 236 protocol (OECD 2013) and adds additional information such as behavior, to the exposure paradigm which strengthens the overall model. Behavioral tracking of larvae following incubation with microplastics resulted in movement that differed from controls (Fig. 5) when larvae were exposed to the highest plastic concentration (0.2 µg/µL). Throughout the first 30 min baseline portion of the behavioral analysis, larvae incubated with microplastic particles appear to travel less distance in both the ZET and GBT assays compared to the control larvae. However, for the GBT larvae exposed to 0.05 and 0.25 µm microplastics, distance traveled is greater than that of the control larvae following the 15-20 min time point. The larvae exposed to 0.05 µm show an increased response to the dark stimulus in comparison with the control in both ZET- and GBT-exposed larvae. The 0.25 µm exposed ZET and GBT larvae travel a similar distance during dark periods of the behavioral assay in comparison to control larvae. The ZET larvae exposed to 0.53, 2.1, 6.02, and 10.2 µm microplastics travel less distance in the dark when compared to the control. This pattern is altered during the GBT assay where, during the dark periods, larvae initially travel a larger distance than the control but then experience a sharp decline in distance traveled by the larvae after 1 min in the dark. After this minute, larvae exposed to 0.53, 2.1, 6.02, and 10.2 µm microplastics travel less than the controls for the remainder of the dark period.

As tracking of larval behavior yielded differences from the control, behavioral data were analyzed for NOEC and LOEC values for each phase of the behavioral assay (Table 1).

**Table 1.** The NOEC and LOEC values resulting from behavioral analysis of zebrafish larvae exposed to micro- and nano-plastic particles during baseline (B), first light–dark (LD), and second light–dark (LD2) transition periods.

	ZET behavioral toxicity (µg/µL)		GBT behavioral toxicity (µg/µL)	
Particle size (µm)	NOEC	LOEC	NOEC	LOEC
0.05	0.001 (B) and 0.1 (LD2)	0.02 (B) and 0.2 (LD2)	0.02 (B)	0.05 (B)
0.25	0.2	–	0.2	–
0.53	0.07 (B) and 0.02 (LD2)	0.1 (B) and 0.05 (LD2)	0.1 (LD)	0.0005 (B and LD2) and 0.2 (LD)
2.1	0.1 (B) and 0.001 (LD)	0.2 (B) and 0.02 (LD)	0.1 (B and LD2)	0.2 (B and LD2)
6.02	0.1 (B)	0.2 (B)	0.1 (B and LD2)	0.2 (B and LD2)
10.2	0.1 (B)	0.2 (B)	0.1 (B and LD2)	0.2 (B and LD2)

Following behavioral analysis, larvae exposed to 0.05 µm particles showed a significant decrease in baseline activity during both ZET and GBT assays (Fig. S9). This effect was seen at concentrations of 0.02 µg/µL in the ZET and at 0.05, 0.07, and 0.1 µg/µL in the GBT. The ZET and GBT baseline NOEC values were 0.001 and 0.02 µg/µL. The baseline LOEC values were then determined to be 0.02 and 0.05 µg/µL for the ZET and GBT assays. A significant increase in response was observed during the second light–dark transition period of the ZET assay at 0.2 µg/µL when compared to control larvae. NOEC and LOEC values for the second ZET light–dark period were then recorded as 0.1 and 0.2 µg/µL.

There were no significant behavioral differences found between control larvae and those exposed to 0.25 µm PS particles. This resulted in a NOEC of 0.2 µg/µL for all time points during the ZET and GBT assays. As the NOEC was the highest tested concentration, no LOEC was determined.

Following exposure to 0.53 µm, larvae showed a significant decrease in total distance traveled in comparison with the control, during the baseline portion of the ZET behavioral assay (Fig. S10). This was seen at 0.1 and 0.2 µg/µL, which resulted in NOEC and LOEC values of 0.07 and 0.1 µg/µL. During the second light–dark period, significant increases in the dark response were seen at 0.05 and 0.07 µg/µL and a significant decrease was seen at 0.2 µg/µL. The corresponding NOEC and LOEC values were 0.02 and 0.05 µg/µL. During the GBT behavioral assay, all significant effects were decreases in total distance traveled and dark response (Fig. S10). This was observed during the baseline at 0.0005, 0.1, and 0.2 µg/µL, during the first light–dark transition at 0.2 µg/µL, as well as during the second light–dark period at 0.0005 and 0.2 µg/µL. The NOEC and LOEC values determined for the first light–dark transition were 0.1 and 0.2 µg/µL. The LOEC value for the second light–dark transition was the lowest concentration tested (0.0005 µg/µL).

Larvae exposed to 2.1 µm microplastics showed a significant decrease in baseline activity compared with control larvae in both the ZET and GBT assays at 0.2 µg/µL (Fig. S11). This resulted in NOEC and LOEC values of 0.1 and 0.2 µg/µL for the ZET, and 0.1 and 0.2 µg/µL for the GBT. During the first ZET light–dark transition, significant increases in larval dark response were observed at 0.02 µg/µL resulting in NOEC and LOEC values of 0.001 and 0.02 µg/µL. Additionally, a significant decrease in response to the dark was recorded during the GBT second light–dark transition at 0.2 µg/µL. For this time period, the NOEC and LOEC values were 0.1 and 0.2 µg/µL.

Larvae exposed to both 6.02 and 10.2 µm particles demonstrated significant decreases in activity during the baseline of both ZET and GBT assays, and significant decreases in dark response during the GBT second light–dark transition at 0.2 µg/µL (Figs S12 and S13). Both ZET and GBT NOEC and LOEC values were then determined to be 0.1 and 0.2 µg/µL.

Overall, significant effects were most often decreases in baseline activity across sizes. Decreases in dark response during the second light–dark period were seen at larger sizes during the GBT assay, and the most variation across assays in LOEC and NOEC values was seen in the smallest tested particle size (0.05 µm). The largest tested particle sizes of 2.1, 6.02, and 10.2 µm all had ZET LOEC values corresponding to the highest tested concentration, and which was also seen during GBT assays for particle sizes larger than 2.1 µm.

## Discussion

### Phenotypic toxicity

Following exposure to the smallest tested size, 0.05 µm, the percent of larvae classified as affected from 72 hpf to 120 hpf appeared to remain constant at most concentrations until the completion of the ZET assay. This was similarly seen in the GBT assay which may suggest that particles of this size begin to affect larvae rapidly after exposure. Conversely, for larvae exposed to 0.53, 6.02, and 10.2 µm particles abnormal phenotypes increased until 96 hpf then decreased after 120 hpf indicating a recovery. Lower toxicity and no significant percentages of affected larvae were seen during the GBT assay of these sizes. As larvae in the ZET assays were incubated with plastic particles throughout development and organogenesis, effects seen in this assay reflect the developmental toxicity of the compound. Conversely, larvae in the GBT assay were exposed to plastic after the completion of the majority of body patterning is complete (72 hpf), and as a result, effects observed show the general toxicity of a compound. Together, these two assays provide different but complimentary information on compound toxicity (Achenbach et al. 2020) which strengthens the overall zebrafish model. In this study, this can be taken to indicate the differences in nano- and micro-plastic toxicity. Here, results suggest that smaller nanoplastics are generally toxic and larger nanoplastics or microplastics are developmentally toxic. Zebrafish have been shown to take up particles via the mouth at later larval stages, such as those seen in the GBT assay, however, toxic phenotypes were observed in the ZET assay, prior to the ability of larvae to ingest particles (Parenti et al. 2019), suggesting that particles were taken up passively (Lu et al. 2016). Concentration ranges were designed based on mass, therefore, more particles were present in the smaller-sized microplastic exposures compared with the larger sizes which may have contributed to the difference in observed toxicity trends. Once taken up by larvae, particles are small enough for transfer from the gastrointestinal tract to other organs (Parenti et al. 2019). These particles can be transferred through endocytosis (Zhang et al. 2009; Clark et al. 2023). Particles larger than 1 µm could also be transported to other organs through phagocytosis (Mayor and Pagano 2007). These respective transfer mechanisms could explain the increasing percent of larvae recorded as affected or dead with increased exposure time for 2.1 µm particles.

The largest particle sizes tested, 6.02 and 10.2 µm, may have been too large to be transported to other organs in the body which resulted in the presence of particles solely within the gut (Mayor and Pagano 2007; Zhang et al. 2009; Clark et al. 2023). The particles are large enough to be excreted out of the gut which could account for the higher incidence of phenotypes seen at earlier exposure time points and the decrease over time. Here and across the literature, the excretion of microplastics within hours has been demonstrated in multiple fish species for various shapes and sizes of microplastics (Grigorakis et al. 2017; Ohkubo et al. 2022).

The decreased incidence of phenotypes seen in the GBT assays, compared with the ZET, may be due to exposure of larvae at a developmental time point where the ability to open their mouth and ingest material is imminent (Parenti et al. 2019). Particles will be more likely to be taken up via ingestion at these time points and progress through the gut (Mayor and Pagano 2007; Zhang et al. 2009; Parenti et al. 2019; Clark et al. 2023). If quickly excreted and cleared from the body, particles may not cause toxic phenotypic effects.

The increased incidence of significant toxic phenotypes seen during the ZET assays for smaller microplastic particles may also be a result of particles accumulating on the chorion (Batel et al. 2018). Particles smaller than the 70-nm diameter chorion pores (Rawson et al. 2000) have the ability to enter the chorion (Xu et al. 2022) and be passively taken up by the larvae during development. Particles larger than this size, may stick to the chorion surfaced thus blocking the pores and interfering with gas exchange (Pereira et al. 2023; Gupta et al. 2024). Both scenarios could cause toxicity such as alterations in hatching timing and success (Luan et al. 2023), as seen in 0.05 µm exposed larvae, or phenotypes such as small heads. During the 0.53, 6.02, and 10.2 µm GBT assays, there are fewer toxic phenotypes observed than the corresponding ZET assay which again supports a reduced gas exchange theory as larvae are exposed to microplastic particles after having hatched from the chorion. However, this requires additional research to determine how plastic interacts with the chorion as plastic was only observed on fragments of the chorion and not on intact chorions prior to larvae hatching.

Additionally, the most common phenotype seen at 72 hpf during the ZET assay across all plastic particle sizes, with the exception of the 0.05 µm, was small head which may be a result of the gas exchange interference mentioned above. Exposure to the 0.53, 6.02, and 10.2 µm particles in the ZET assay resulted in the large yolk phenotype being most commonly observed at 96 hpf. This may suggest potential impediment of yolk sac resorption due to microplastic exposure which has been previously recorded in zebrafish larvae (Rabezanahary et al. 2023). At 96 hpf, scoliosis was the most prominent phenotype in the 2.1 µm exposed larvae and tail and column deformities have been previously recorded in zebrafish larvae exposed to larger-sized plastic particles (De Marco et al. 2022). Most other particle sizes resulted in an uninflated swim bladder as the most recorded phenotype. This may be a result of altered larval behavior that impeded the inflation of the swim bladder or a physiological effect that prevented swim bladder inflation.

During the GBT exposure, the three largest microplastic particle sizes saw large yolk as the most commonly observed phenotype at 96 hpf similar to the ZET assay at the same time point. This phenotype may be caused by a general delay in developmental progression, however, there are no other phenotypes recorded that would support this. Large yolks may also have been caused by particles accumulating in the gastrointestinal tract thus reducing the reuptake of nutrients from the yolk sac. Microplastic particles have been previously seen to accumulate within the gastrointestinal tract (Chen et al. 2020) and be cleared quickly via excretion (Yu et al. 2022) which may explain the transient nature of this phenotype. This is supported by studies that have shown that waterborne microplastic exposure results in higher numbers of plastic particles taken up by larvae and a shorter residence time compared with foodborne exposure (Yu et al. 2022). Nanoplastics have been observed within larval vasculature (Dai et al. 2023) which may also have reduced the amount of nutrients from the yolk reaching necessary organs. Plastic within the gastrointestinal tract may also result in the overproduction of mucous in the gut as well as the gills (Limonta et al. 2019) which have been an observed site of plastic particle accumulation (Batel et al. 2018; Limonta et al. 2019; Chen et al. 2020). Particles can accumulate on the mucous layer on the gills (Batel et al. 2018), potentially inducing the mentioned hypersecretion as the larvae attempt to shed the mucosal layer with the particles.

### Behavioral toxicity

Overall, microplastic exposure decreased the baseline distance traveled by larvae. Larvae exposed to 0.05 and 2.1 µm plastic particles saw an increase in activity in the dark during the transitions, whereas larvae exposed to the larger particles (6.02 and 10.2 µm) showed a decrease in activity compared with the control larvae. A notable exception to this is the larvae exposed to 0.53 µm particles (ZET), which showed a decrease in dark activity at the highest exposure concentration, and an increase in activity at two mid-range concentrations (0.05 and 0.07 µg/µL).

This change in activity pattern appears to occur at 0.53 µm nanoplastics, where both increased and decreased activity in the dark is seen in the ZET and only decreased activity in the dark is seen in the GBT. With larger particle sizes, the pattern changes to a general decrease in baseline and dark activity for both ZET and GBT periods. This suggests that microplastics have the potential to affect both baseline and stress response behaviors in larval zebrafish. The decreased baseline activity of larvae seen across all sized microplastics has been previously documented for both micro- and nano-plastics (Chen et al. 2017, 2020; Qiang and Cheng 2019; Cormier et al. 2022; Feng et al. 2022). Increased activity in the dark seen in smaller particle exposures has been previously documented following exposure to PS microplastics smaller than 1 µm in diameter (Limonta et al. 2019). It has been suggested that microplastic exposure may affect the visual capabilities of the larvae and their recognition of darkness as a stimulus (Rabezanahary et al. 2023). As microplastic particles were seen on eye surfaces in larvae exposed to 0.05, 0.25, and 0.53 µm microplastic particles, this may have impaired their vision thus limiting their ability to respond to external stressors.

Overall, this study demonstrates that exposure to micro- and nano-plastic particles may result in adverse effects on larval fish. Morphological abnormalities, such as those seen in this study, may inhibit normal function of fish in the environment for instance, moving throughout the water column if a functional swim bladder is absent or survival if yolk sac reabsorption is hindered. This may be compounded with effects on behavior that may influence the organism’s ability to escape predation or identify food sources. Combined, these individual effects could impact the population as a whole if survival decreases, or the community, as survival of one population may have cascading repercussions on other organisms within the food web.

## Conclusion

Micro- and nano-plastics have shown the potential to be taken up by larval zebrafish as early as 72 hpf, and result in particle presence within various organs including the gastrointestinal tract and gills. All tested particle sizes showed evidence of being excreted from the larvae (0.05, 0.25, 0.53, 2.1, 6.02, and 10.2). The toxicity of microplastic particles appears to increase as particle size decreases, and recovery from exposure seems possible at the larger particle sizes tested (0.53, 6.02, and 10.2 µm). Both micro- and nano-plastics appear to be potentially neurotoxic through affecting both baseline and stress response behaviors of larval zebrafish. Overall, micro- and nano-plastics show the potential to be developmentally and generally toxic, respectively. Future studies should examine subphenotypic effects, such as gene expression, in order to understand the effects of plastic particles on model organisms. Chronic exposures have been shown to be advantageous in understanding the long-term effects of plastic on reproductive success, fitness, and impact on F1 generations (Zuo et al. 2021; Zhang et al. 2024; Ren et al. 2024; Zeng et al. 2024; Pitt et al. 2018). Future work would be required to extrapolate the impact of plastic particles at the population level and could provide a glimpse into the potential impacts on the environment.

## Supplementary Material

kfaf015_Supplementary_Data
