# Supplementary material for: Evaluation of phenotypic and behavioral toxicity of micro- and nano-plastic polystyrene particles in larval zebrafish (Danio rerio)
Source: Toxicol Sci. 2025 Feb 8;205(1):154–65. doi: 10.1093/toxsci/kfaf015 (PMC12038248; doi:10.1093/toxsci/kfaf015)
Supplement: kfaf015_Supplementary_Data [file kfaf015_supplementary_data.docx]

**Title:** Evaluation of phenotypic and behavioral toxicity of micro- and nano-plastic polystyrene particles in larval zebrafish (*Danio rerio*)

**Authors:** Bailey Levesque ^a^, Sabahudin Hrapovic ^b^, Fabrice Berrué ^a^, Anja Vogt ^c^, Lee Ellis ^a^, Ludovic Hermabessiere ^a*^

^a^ National Research Council Canada, Aquatic and Crop Resource Development Research Centre, 1411 Oxford Street, Halifax, NS B3H 3Z1, Canada

^b^ National Research Council Canada, Aquatic and Crop Resource Development Research Centre, 6100 Royalmount Avenue, Montréal, QC H4P 2R2, Canada

^c^ National Research Council Canada, Aquatic and Crop Resource Development Research Centre, 550 University Avenue, Charlottetown, PEI C1A 4P3, Canada

* Corresponding author: Ludovic.Hermabessiere@nrc-cnrc.gc.ca

**Supplementary Information**

**Supplementary Material and Methods**

*Particle Fluorophore Leaching*

A square well flat bottomed 96- well polystyrene plate was filled as described in the *Phenotypic Toxicity - Fish Embryo Toxicity Assay* methods however, no larvae were added. The plate was incubated under the same conditions as exposure plates for 120 hours. Following incubation, liquid was filtered through a 0.2 µm PES filter (Fisherbrand, Fisher Scientific, New Hampshire, USA) to remove particles and the filtered liquid was used for experimentation. The fluorescence of the leachate was then measured using a plate reader (Biotek Cytation 5, Agilent Technologies, CA, USA) at an excitation/emission wavelength of 500/560 nm. Samples were run in triplicate.

Table S1 Size range, mean diameter, and catalog numbers associated with all polystyrene microplastic particles used.

| **Theorical Size Range (µm)** | **Mean theorical diameter (µm)** | | **Catalog Number** | |
| --- | --- | --- | --- | --- |
| 0.04-0.06 | 0.04 | FP-00556-2 | |  |
| 0.1-0.3 | 0.25 | FP-0256-2 | |  |
| 0.4-0.6 | 0.53 | FP-0556-2 | |  |
| 1.7-2.2 | 2.1 | FP-2056-2 | |  |
| 5.0-7.9 | 6.02 | FP-6056-2 | |  |
| 10.0-14.0 | 10.2 | FP-10056-2 | |  |

Table S2 Advertised size range, measured size, and zeta potential of polystyrene microplastic spheres measured by Dynamic Light Scattering.

| **Size Range (µm)** | **Mean Measured Diameter (µm) ±**  **Standard Deviation** | | **Mean Zeta Potential (mV) ± Standard Deviation** | |
| --- | --- | --- | --- | --- |
| 0.04-0.06 | 0.0506 ± 0.0004 | -40.5 ± 1.04 | |  |
| 0.1-0.3 | 0.2421 ± 0.00467 | -44 ± 0.265 | |  |
| 0.4-0.6 | 0.4838 ± 0.00566 | -44.9 ± 0.451 | |  |
| 1.7-2.2 | 3.764 ± 0.1634 | -64.1 ± 1.23 | |  |
| 5.0-7.9 | 5.009 ± 0.6728 | -31.8 ± 3.46 | |  |
| 10.0-14.0 | 11.36 ± 1.078 | -76.6 ± 1.66 | |  |

Table S3 The calculated concentration of micro- and nano-plastic spheres in particles per millilitre compared to the nominal dose used in experimentation. Calculations were based off of DLS measurements (Table S2).

| **Exposure Concentration (µg/µL)** | **Particle Size (µm)** | | | | | |
| --- | --- | --- | --- | --- | --- | --- |
|  | **0.05** | **0.25** | **0.53** | **2.1** | **6.02** | **10.2** |
| 0.0005 | 7,019,894,000 | 64,091,000 | 8,031,000 | 17,000 | 7,000 | 1,000 |
| 0.001 | 14,039,787,000 | 128,182,000 | 16,063,000 | 34,000 | 14,000 | 1,000 |
| 0.02 | 280,795,746,000 | 2,563,644,000 | 321,251,000 | 682,000 | 289,000 | 25,000 |
| 0.05 | 701,989,365,000 | 6,409,110,000 | 803,127,000 | 1,705,000 | 724,000 | 62,000 |
| 0.07 | 982,785,110,000 | 8,972,754,000 | 1,124,378,000 | 2,388,000 | 1,013,000 | 87,000 |
| 0.1 | 1,403,978,729,000 | 12,818,220,000 | 1,606,255,000 | 3,411,000 | 1,447,000 | 124,000 |
| 0.2 | 2,807,957,458,000 | 25,636,439,000 | 3,212,510,000 | 6,822,000 | 2,895,000 | 248,000 |


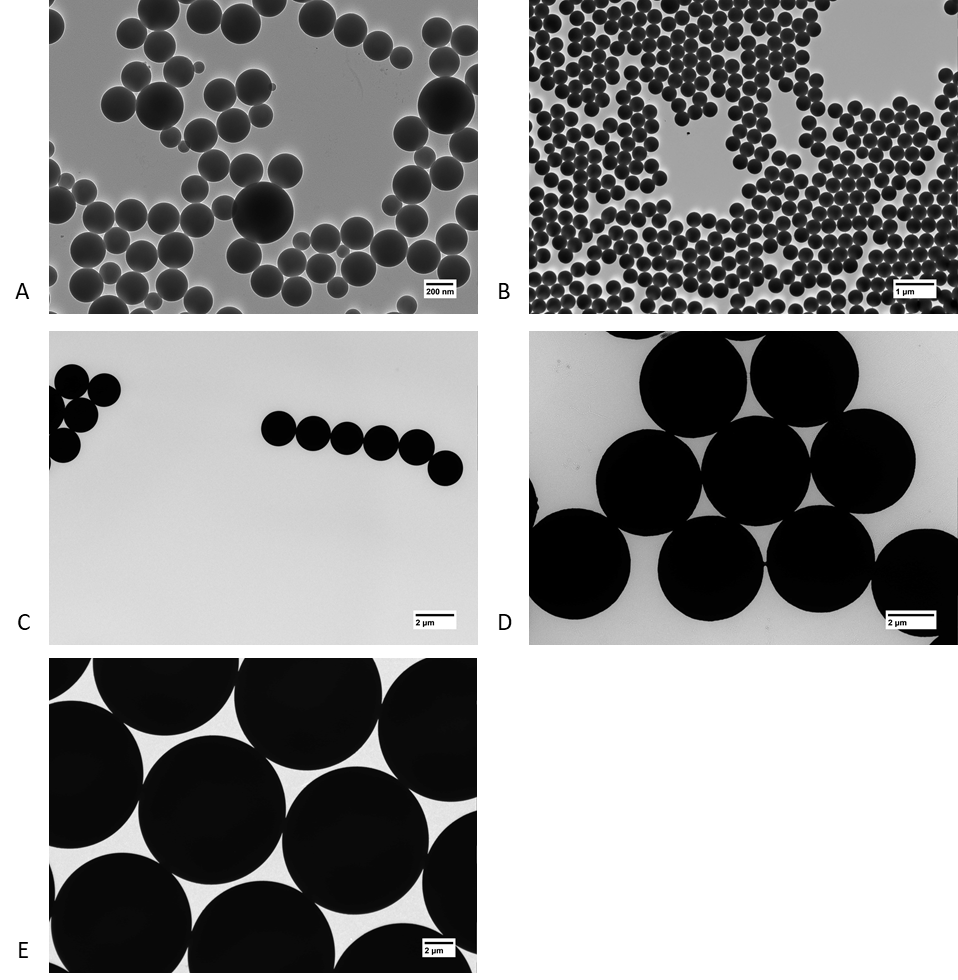


Figure S1 TEM images of polystyrene microspheres with diameters of 0.25 (A), 0.53 (B), 2.1 (C), 6.02 (D), and 10.2 µm (E).


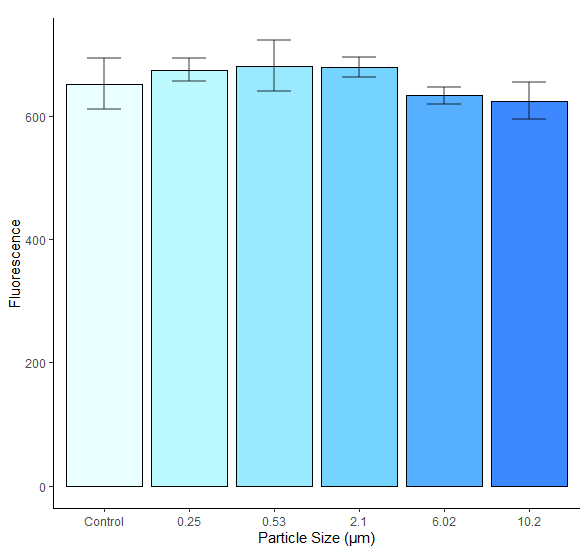


Figure S2 Mean (+ standard deviation) fluorescence measured for microplastic particles incubated for 5 days in HE3 media and filtered. No significant difference was determined at p < 0.05.

Table S4 Location of micro- and nano- plastic particle accumulation as seen through fluorescent microscopy following ZET and GBT exposures to 6 different sized particles.

| Location of Accumulation | ZET | | | | | | GBT | | | | | |
| --- | --- | --- | --- | --- | --- | --- | --- | --- | --- | --- | --- | --- |
|  | 0.05 µm | 0.25 µm | 0.53 µm | 2.1 µm | 6.02 µm | 10.2 µm | 0.05 µm | 0.25 µm | 0.53 µm | 2.1 µm | 6.02 µm | 10.2 µm |
| Gastrointestinal Tract | ✓ | ✓ | ✓ | ✓ | ✓ | ✓ | ✓ | ✓ | ✓ | ✓ | ✓ | ✓ |
| Gills | ✓ | ✓ | ✓ |  |  |  | ✓ | ✓ | ✓ |  |  |  |
| Mouth | ✓ | ✓ |  | ✓ | ✓ | ✓ | ✓ | ✓ |  | ✓ | ✓ | ✓ |
| Esophagus | ✓ | ✓ | ✓ | ✓ | ✓ | ✓ |  |  | ✓ | ✓ |  | ✓ |
| Dermal Tissue | ✓ |  |  |  |  |  | ✓ | ✓ |  |  |  |  |
| Eyes |  |  | ✓ |  |  |  | ✓ | ✓ | ✓ |  |  |  |


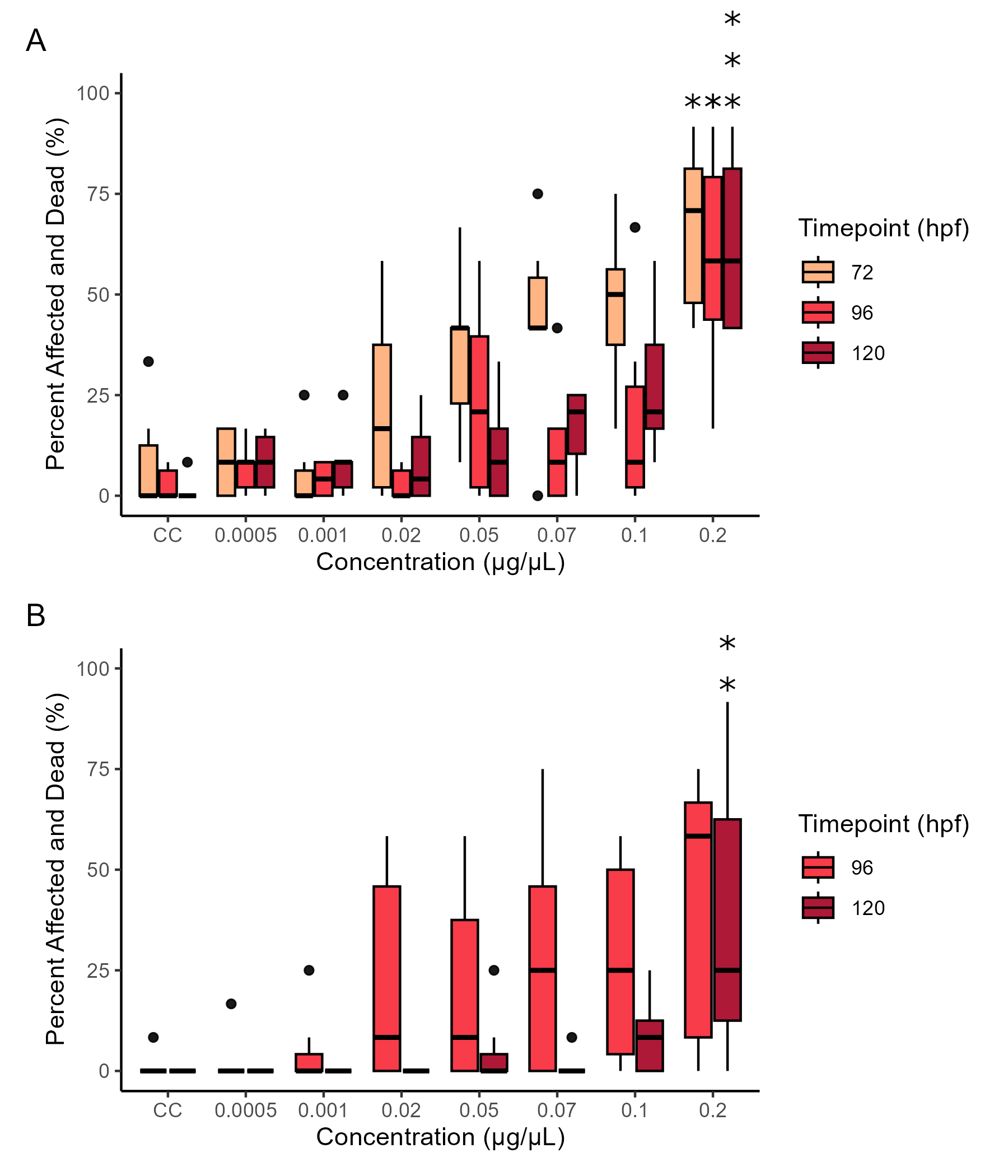


Figure S3 Percent of affected and dead zebrafish larvae (*Danio rerio*) (n = 72 - 84) exposed to 0.05 µm microplastic particles tested during the ZET (A) and GBT (B) assays at multiple microplastic concentrations (in µg/µL) and timepoints. Significant differences between the control (CC) and plastic concentrations calculated using a non-parametric Kruskal-Wallis test with a Dunn’s multiple comparisons test (*: p<0.05; **: p<0.01; ***: p<0.001) are noted. Boxplot bottom line: lower quartile; boxplot midline: median; boxplot upper line: last quartile; bottom of whisker: minimum calculated value; upper of whisker: maximum calculated value; point: outliers.


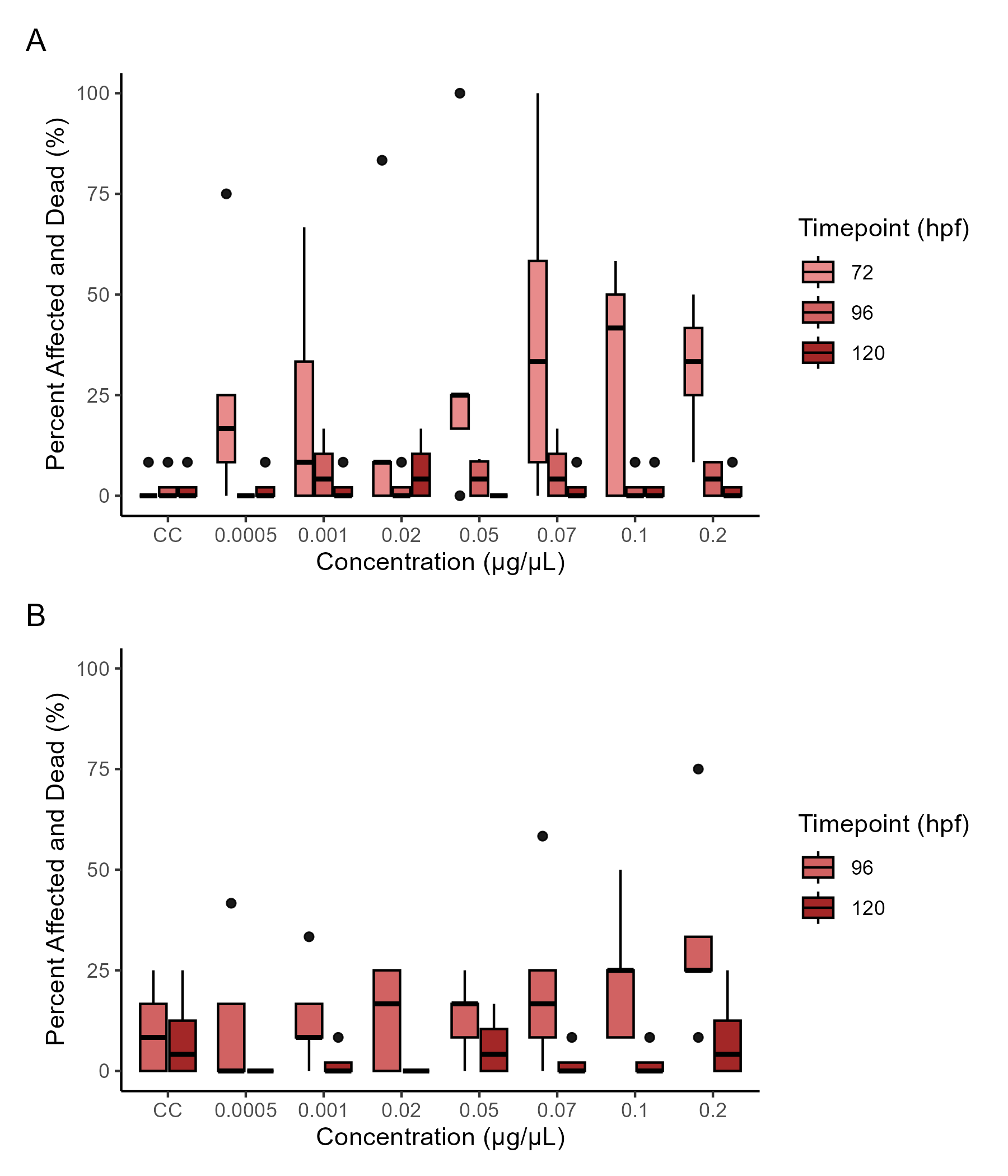


Figure S4 Percent of affected and dead zebrafish larvae (*Danio rerio*) (n = 47 - 60) exposed to 0.25 µm microplastic particles tested during the ZET (A) and GBT (B) assays at multiple microplastic concentrations (in µg/µL) and timepoints. Significant differences between the control (CC) and plastic concentrations calculated using a non-parametric Kruskal-Wallis test with a Dunn’s multiple comparisons test (*: p<0.05; **: p<0.01; ***: p<0.001) are noted. Boxplot bottom line: lower quartile; boxplot midline: median; boxplot upper line: last quartile; bottom of whisker: minimum calculated value; upper of whisker: maximum calculated value; point: outliers.


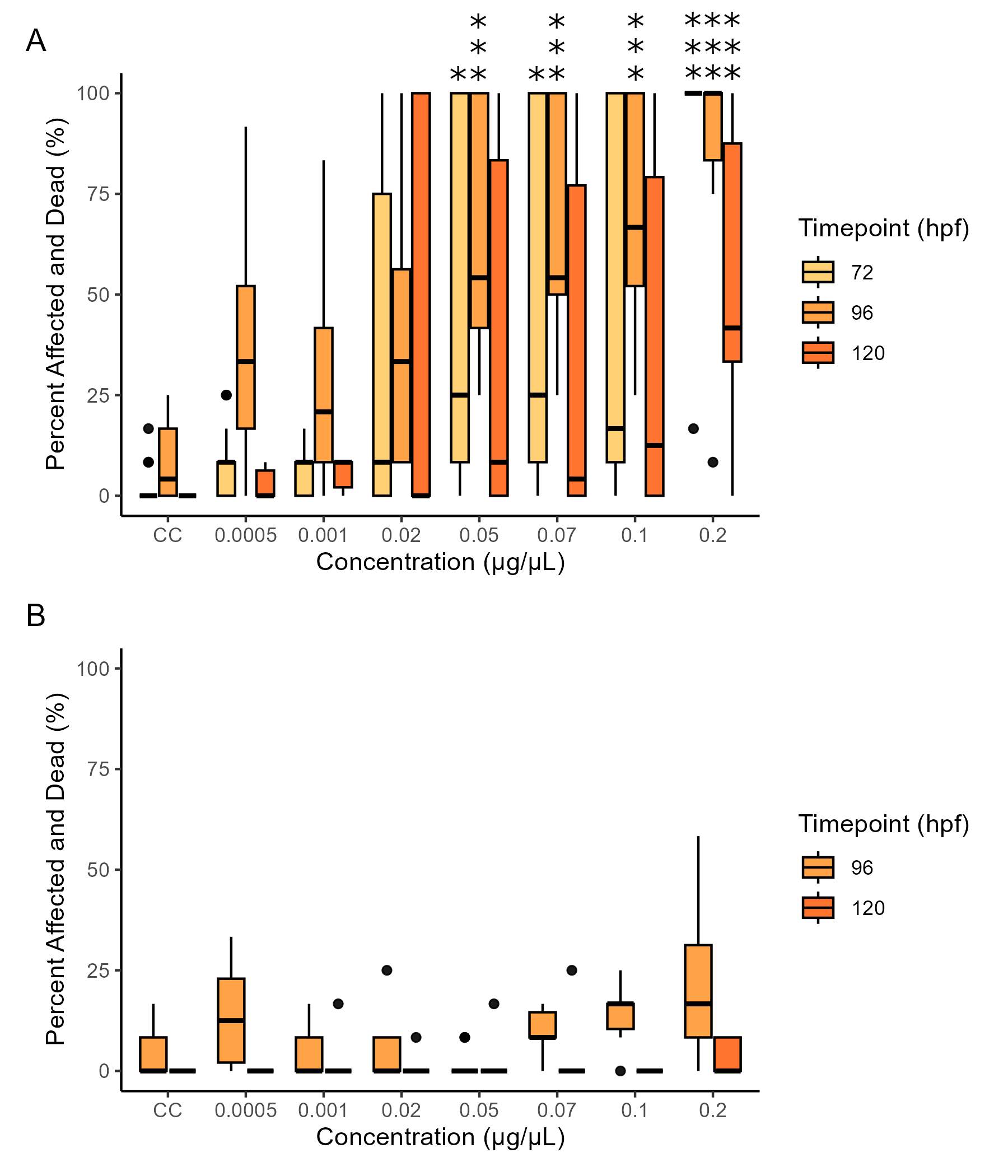
 Figure S5 Percent of affected and dead zebrafish larvae (*Danio rerio*) (n = 108 - 156) exposed to 0. 53 µm microplastic particles tested during the ZET (A) and GBT (B) assays at multiple microplastic concentrations (in µg/µL) and timepoints. Significant differences between the control (CC) and plastic concentrations calculated using a non-parametric Kruskal-Wallis test with a Dunn’s multiple comparisons test (*: p<0.05; **: p<0.01; ***: p<0.001) are noted. Boxplot bottom line: lower quartile; boxplot midline: median; boxplot upper line: last quartile; bottom of whisker: minimum calculated value; upper of whisker: maximum calculated value; point: outliers.


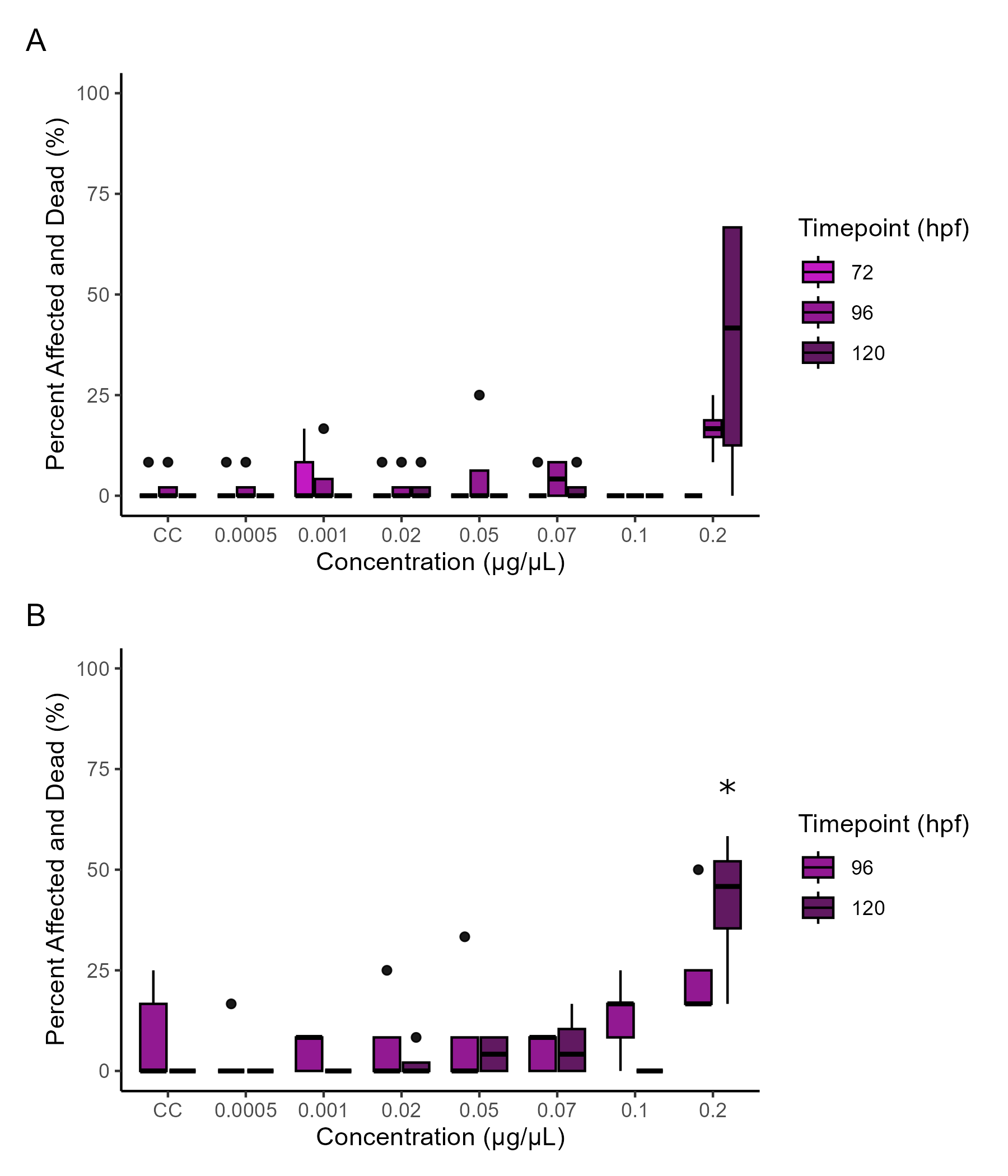
 Figure S6 Percent of affected and dead zebrafish larvae (*Danio rerio*) (n = 48 - 60) exposed to 2.1 µm microplastic particles tested during the ZET (A) and GBT (B) assays at multiple microplastic concentrations (in µg/µL) and timepoints. Significant differences between the control (CC) and plastic concentrations calculated using a non-parametric Kruskal-Wallis test with a Dunn’s multiple comparisons test (*: p<0.05; **: p<0.01; ***: p<0.001) are noted. Boxplot bottom line: lower quartile; boxplot midline: median; boxplot upper line: last quartile; bottom of whisker: minimum calculated value; upper of whisker: maximum calculated value; point: outliers.


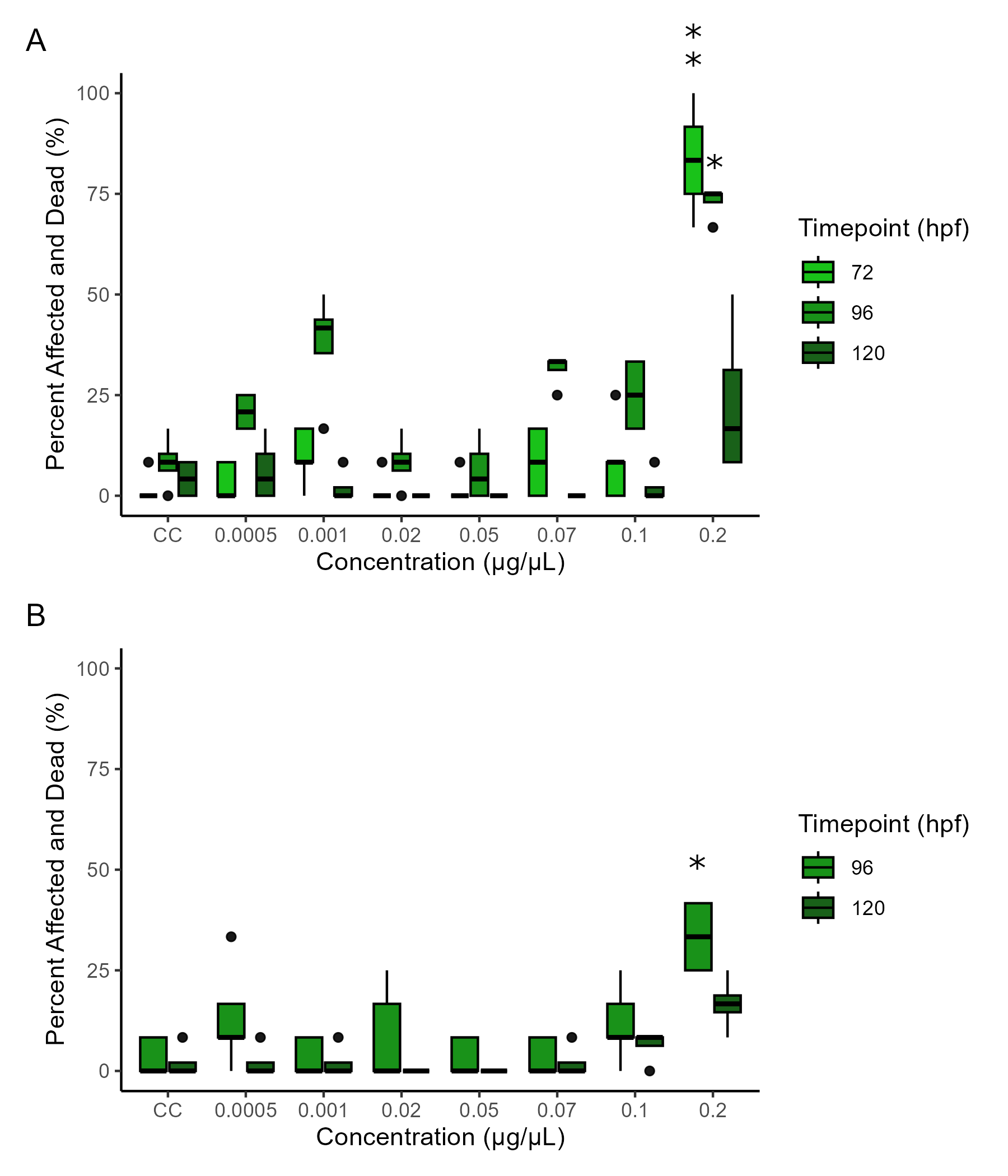
 Figure S7 Percent of affected and dead zebrafish larvae (*Danio rerio*) (n = 48 - 60) exposed to 6.02 µm microplastic particles tested during the ZET (A) and GBT (B) assays at multiple microplastic concentrations (in µg/µL) and timepoints. Significant differences between the control (CC) and plastic concentrations calculated using a non-parametric Kruskal-Wallis test with a Dunn’s multiple comparisons test (*: p<0.05; **: p<0.01; ***: p<0.001) are noted. Boxplot bottom line: lower quartile; boxplot midline: median; boxplot upper line: last quartile; bottom of whisker: minimum calculated value; upper of whisker: maximum calculated value; point: outliers.


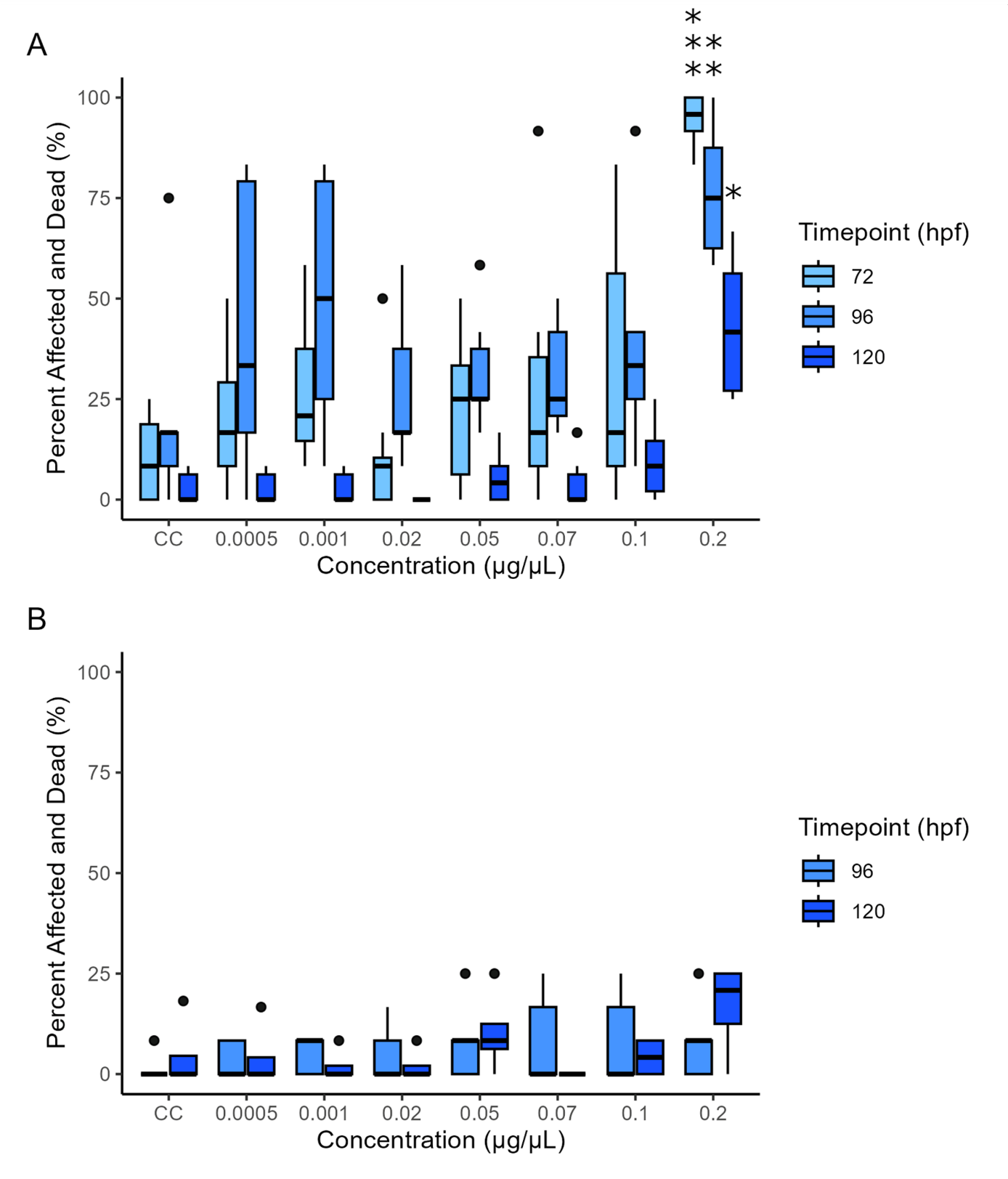
 Figure S8 Percent of affected and dead zebrafish larvae (*Danio rerio*) (n = 47 - 96) exposed to 10.2 µm microplastic particles tested during the ZET (A) and GBT (B) assays at multiple microplastic concentrations (in µg/µL) and timepoints. Significant differences between the control (CC) and plastic concentrations calculated using a non-parametric Kruskal-Wallis test with a Dunn’s multiple comparisons test (*: p<0.05; **: p<0.01; ***: p<0.001) are noted. Boxplot bottom line: lower quartile; boxplot midline: median; boxplot upper line: last quartile; bottom of whisker: minimum calculated value; upper of whisker: maximum calculated value; point: outliers.

Table S5 Most commonly observed phenotype in affected larvae at each assay timepoint following exposure to micro- and nano- plastic particles

| Particle Size (µm) | FET Assay Timepoint (hpf) | | | GBT Assay Timepoint (hpf) | |
| --- | --- | --- | --- | --- | --- |
|  | 72 | 96 | 120 | 96 | 120 |
| 0.05 | Unhatched | Light Colour | Scoliosis | Light Colour | No Swim Bladder |
| 0.25 | Small Head | Light Colour | No Swim Bladder | Scoliosis | Scoliosis |
| 0.53 | Small Head | Large Yolk | No Swim Bladder | Scoliosis | No Swim Bladder |
| 2.1 | Small Head | Scoliosis | No Swim Bladder | Large Yolk | No Swim Bladder |
| 6.04 | Small Head | Large Yolk | No Swim Bladder | Large Yolk | No Swim Bladder |
| 10.1 | Small Head | Large Yolk | Large Yolk | Large Yolk | No Swim Bladder |


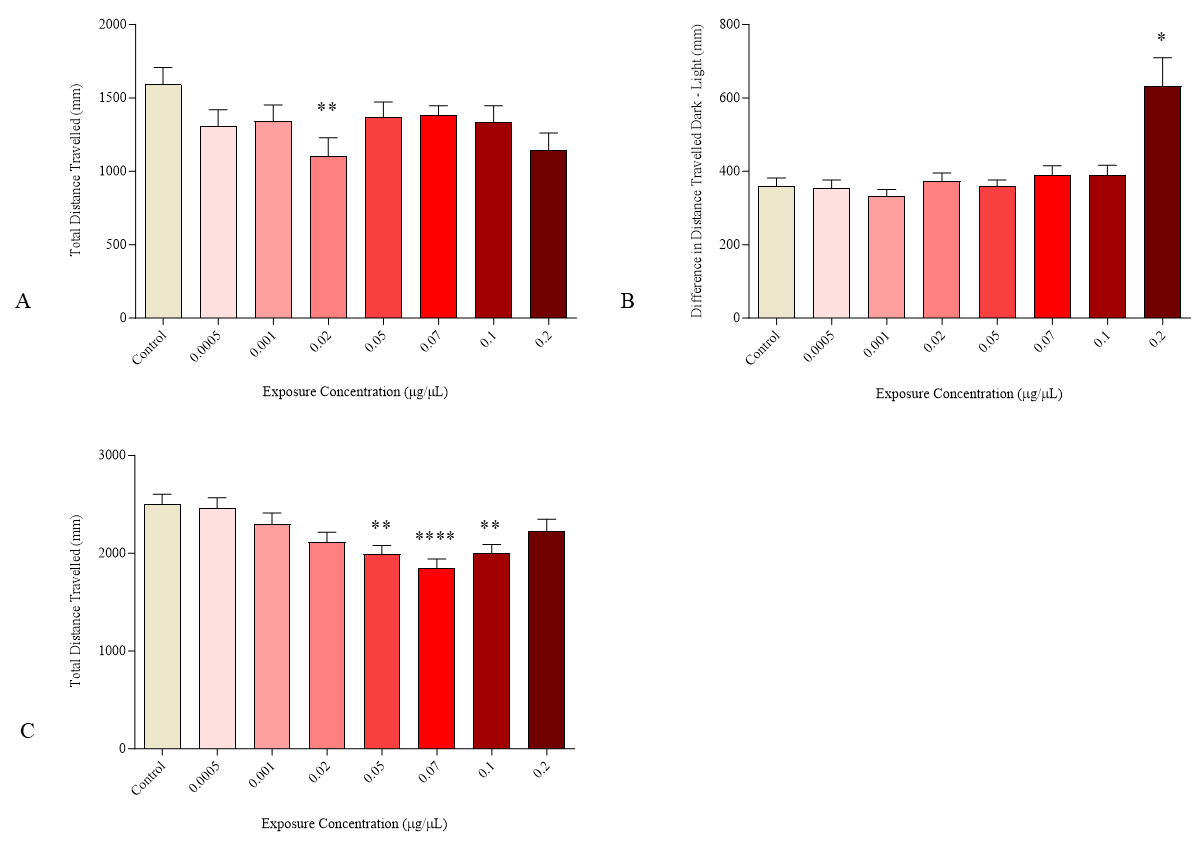


Figure S9 Mean distance (+ Standard Error of the Mean) travelled by larvae exposed to 0.05 µm microplastic particles at 120 hpf of the FET (n = 6 - 33) during the baseline and second line dark periods of the assay as well as the baseline period of the GBT (n = 51 - 72) assay. Significance determined at p < 0.05 (*: p<0.05; **: p<0.01; ***: p<0.001).


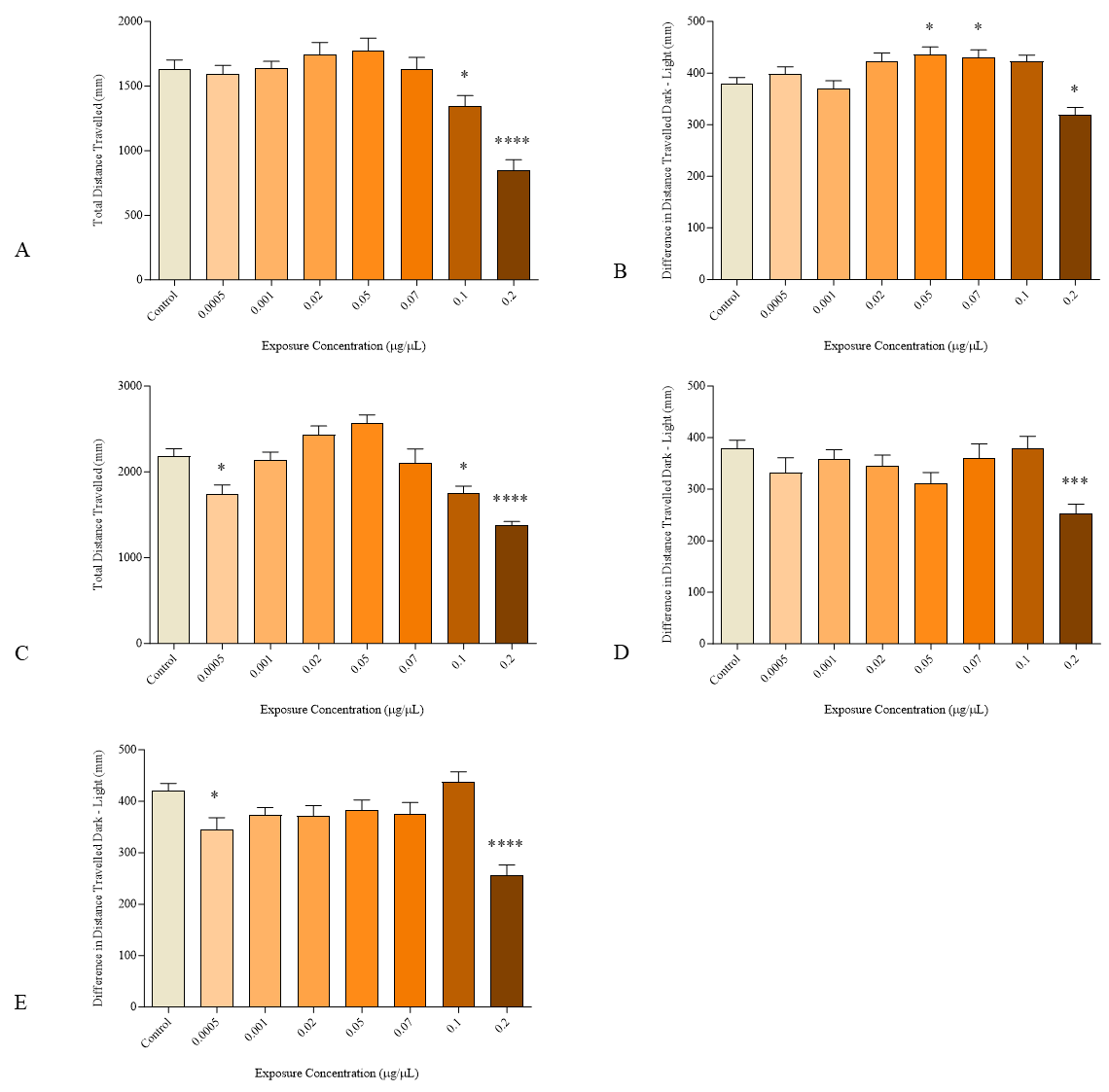


Figure S10 Mean distance (+ Standard Error of the Mean) travelled by larvae exposed to 0.53 µm microplastic particles at 120 hpf during the baseline (A) and second light dark transition (B) of the FET (n = 44 - 72) assay. The baseline (C), first light- dark transition (D), and second light- dark transition (E) data for the GBT (n = 34 - 36) assay are also included. Significance determined at p < 0.05 (*: p<0.05; **: p<0.01; ***: p<0.001).

**
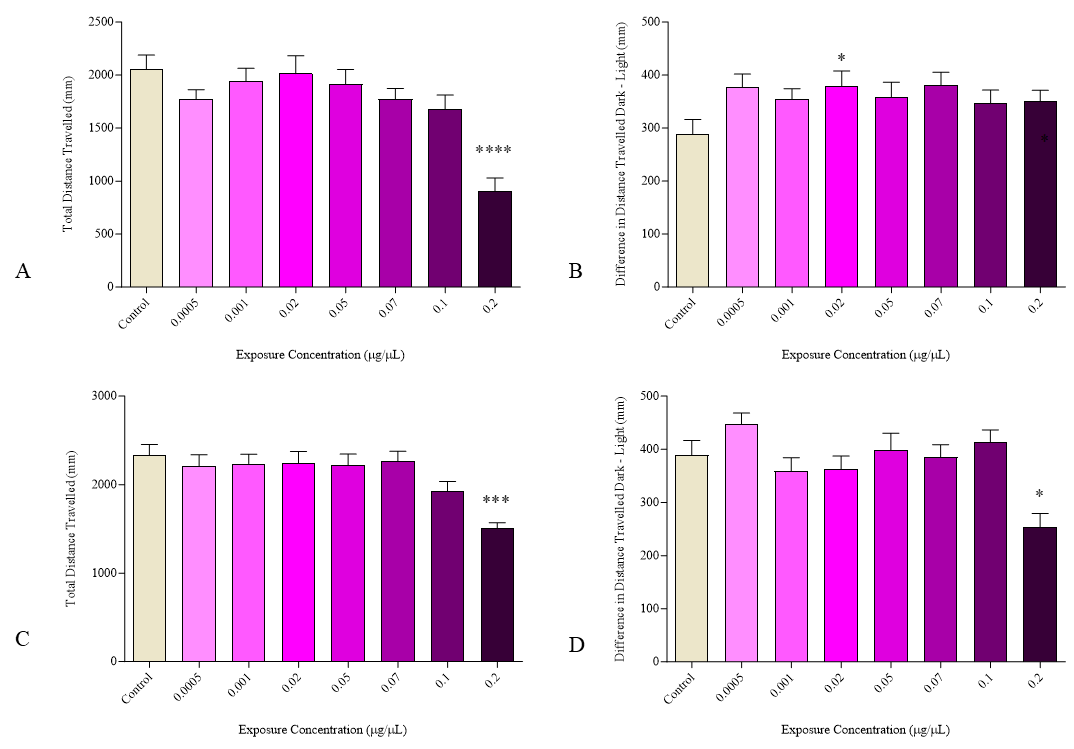
**

Figure S11 Mean distance (+ Standard Error of the Mean) travelled by larvae exposed to 2.1 µm microplastic particles during the baseline (A) and first light dark transition (B) periods of the FET (n = 23 - 36) behavioural assay. Behaviour during the baseline (C) and second light dark transition of the GBT (n = 15 - 36) assay are shown. Significance was determined at p < 0.05 (*: p<0.05; **: p<0.01; ***: p<0.001).

**
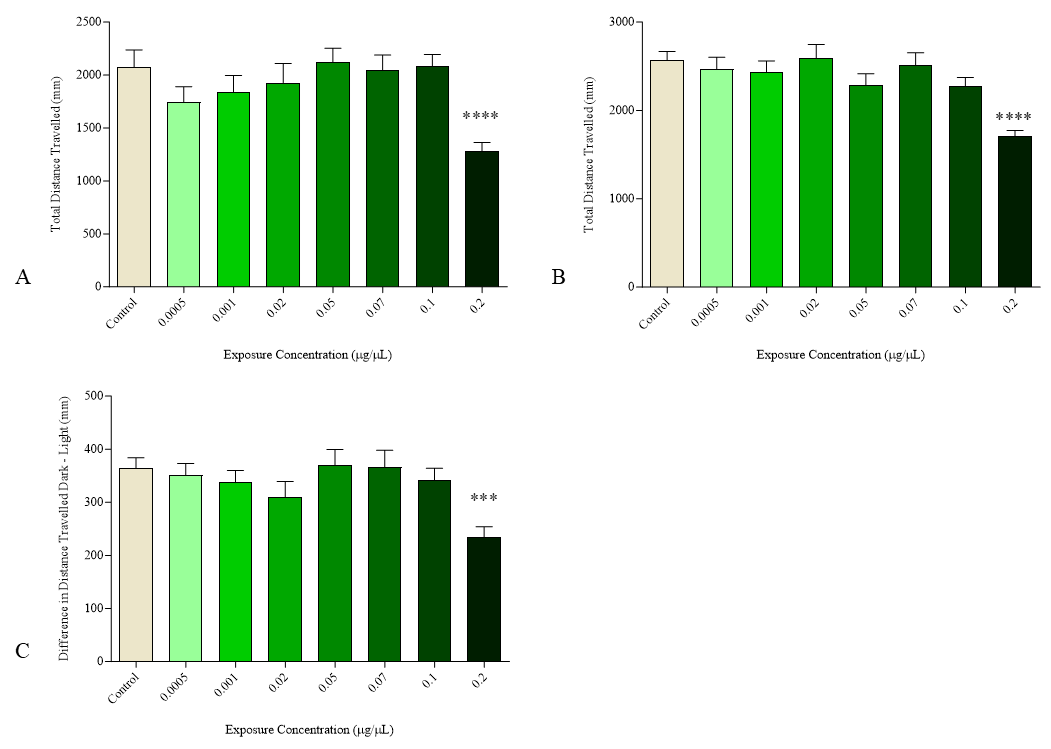
**

Figure S12 Mean distance (+ Standard Error of the Mean) travelled by larvae during the FET (n = 31 - 36) baseline (A) and GBT (n = 30 - 36) baseline (B) and second light dark period (C) portions of the 6.02 µm microplastic behavioural assays. Significance was determined at p < 0.05 (*: p<0.05; **: p<0.01; ***: p<0.001).

**
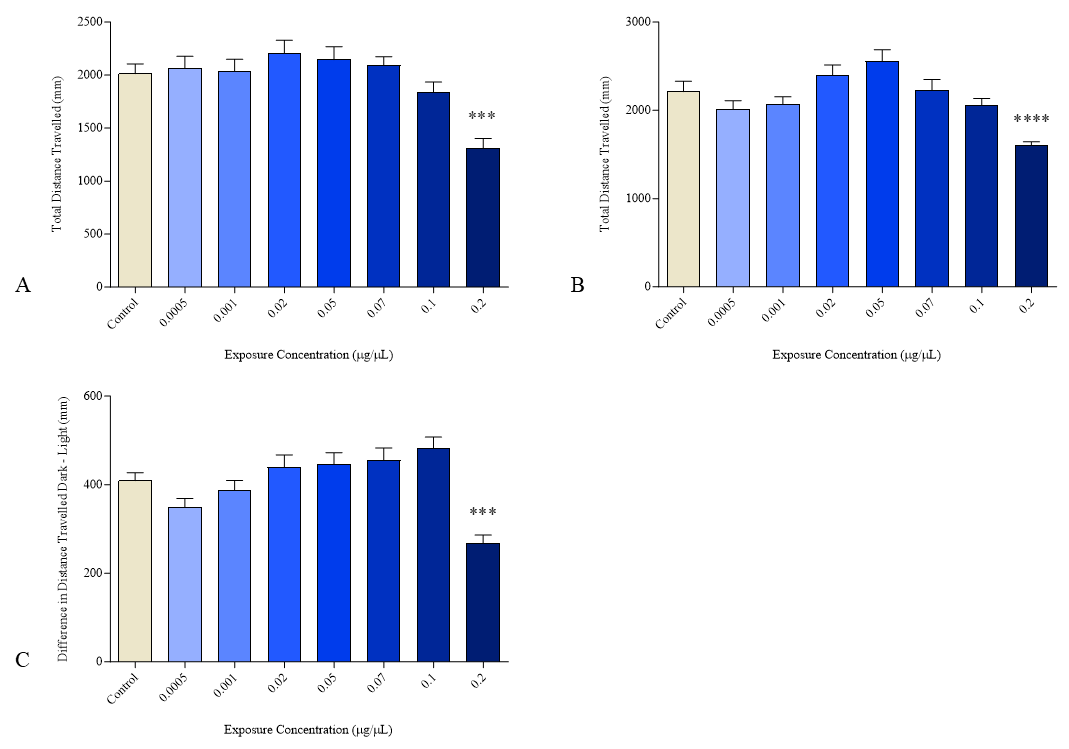
**

Figure S13 Mean distance (+ Standard Error of the Mean) travelled by larvae exposed to 10.2 µm microplastic particles at 120 hpf during the FET (n = 35 - 60) baseline (A) and GBT (n = 31 - 36) baseline (B) and second light dark transition (C) periods. Significance was determined at p < 0.05 (*: p<0.05; **: p<0.01; ***: p<0.001).
